# Conjugative transfer of multi-drug resistance IncN plasmids from environmental waterborne bacteria to *Escherichia coli*

**DOI:** 10.3389/fmicb.2022.997849

**Published:** 2022-10-26

**Authors:** Jessica Guzman-Otazo, Enrique Joffré, Jorge Agramont, Nataniel Mamani, Jekaterina Jutkina, Fredrik Boulund, Yue O. O. Hu, Daphne Jumilla-Lorenz, Anne Farewell, D. G. Joakim Larsson, Carl-Fredrik Flach, Volga Iñiguez, Åsa Sjöling

**Affiliations:** ^1^Institute of Molecular Biology and Biotechnology, Universidad Mayor de San Andrés, La Paz, Bolivia; ^2^Department of Microbiology, Tumor and Cell Biology, Karolinska Institutet, Stockholm, Sweden; ^3^Centre for Translational Microbiome Research, Karolinska Institutet, Stockholm, Sweden; ^4^Department of Infectious Diseases, Institute of Biomedicine, Sahlgrenska Academy, University of Gothenburg, Göteborg, Sweden; ^5^Centre for Antibiotic Resistance Research (CARe), University of Gothenburg, Göteborg, Sweden; ^6^Department of Chemistry and Molecular Biology, University of Gothenburg, Göteborg, Sweden

**Keywords:** waterborne bacteria, horizontal gene transfer, multi-drug resistance, *Escherichia coli*, IncN plasmid, copper sulfate, zinc sulfate, conjugative plasmid transfer

## Abstract

Watersheds contaminated with municipal, hospital, and agricultural residues are recognized as reservoirs for bacteria carrying antibiotic resistance genes (ARGs). The objective of this study was to determine the potential of environmental bacterial communities from the highly contaminated La Paz River basin in Bolivia to transfer ARGs to an *Escherichia coli* lab strain used as the recipient. Additionally, we tested ZnSO_4_ and CuSO_4_ at sub-inhibitory concentrations as stressors and analyzed transfer frequencies (TFs), diversity, richness, and acquired resistance profiles. The bacterial communities were collected from surface water in an urban site close to a hospital and near an agricultural area. High transfer potentials of a large set of resistance factors to *E. coli* were observed at both sites. Whole-genome sequencing revealed that putative plasmids belonging to the incompatibility group N (IncN, IncN2, and IncN3) were predominant among the transconjugants. All IncN variants were verified to be mobile by a second conjugation step. The plasmid backbones were similar to other IncN plasmids isolated worldwide and carried a wide range of ARGs extensively corroborated by phenotypic resistance patterns. Interestingly, all transconjugants also acquired the class 1 integron *intl1*, which is commonly known as a proxy for anthropogenic pollution. The addition of ZnSO_4_ and CuSO_4_ at sub-inhibitory concentrations did not affect the transfer rate. Metal resistance genes were absent from most transconjugants, suggesting a minor role, if any, of metals in the spread of multidrug-resistant plasmids at the investigated sites.

## Introduction

During the last decades, infections caused by antibiotic-resistant bacteria have escalated worldwide, positioning antibiotic resistance as one of the worst health-threatening problems for mankind (Fauci and Marston, 2014; Petchiappan and Chatterji, 2017). Insufficient hygiene, use, misuse, and over-use of antibiotics, and the release of selective agents to the environment have further potentiated the occurrence and dispersion of antibiotic resistance (Andersson and Hughes, 2014; Aubertheau et al., 2017; Tan et al., 2018). Water bodies and soils receive discharges of pathogenic and non-pathogenic bacteria, antibiotics, biocides, metals, and other chemical residues from hospital and community settings as well as from agriculture and animal husbandry (Andersson and Hughes, 2014; Singer et al., 2016; Pal et al., 2017). Therefore, contaminated water bodies and aquatic sediments may facilitate the emergence and dissemination of pollutant- and antibiotic-resistant bacteria (ARB) (Finley et al., 2013; Marti et al., 2014; Bengtsson-Palme et al., 2018; Larsson and Flach, 2022). In Bolivian cities such as La Paz, the lack of wastewater treatment and discharges from hospitals, industries, and households directly into the La Paz River basin have significantly contributed to the pollution of river water, which is often used for irrigation of agricultural areas located downstream. Several studies have found large numbers of pathogenic and resistant bacteria isolated from river water, soil, and vegetable samples from the La Paz River basin (Poma et al., 2016; Guzman-Otazo et al., 2019; Medina et al., 2021).

Antibiotic-resistant bacterias can emerge by mutations in target genes or by acquiring genes by horizontal gene transfer (HGT). Bacterial conjugation is one of the most common HGT mechanisms for antibiotic resistance dispersion (Andersson and Hughes, 2017). Moreover, multi-drug resistance in bacteria has evolved at least partly by co-selection through co- and cross-resistance mechanisms (multiple resistance genes within a mobile genetic element (MGE) versus the presence of resistance genes with a broad substrate range) (Marti et al., 2014; Pal et al., 2014, 2015).

Several stressors affecting transfer of ARGs have been identified; for instance, different kinds of metals such as Cu, Zn, and Hg might influence the occurrence and mobilization of ARGs in the environment since antibiotic and metal resistance genes often co-exist in the same MGE (Ji et al., 2012; Pal et al., 2015). These metals can be found in downstream water from mining and industrial areas (Rogozin and Gavrilkina, 2008; Agramont et al., 2020). In Bolivia, lakes and rivers located upstream of the La Paz River basin have been shown to be highly impacted by acid mining drainage discharges due to the intensive mining activity carried out during the last century (Agramont et al., 2020). The transfer of antibiotic resistance from bacterial communities in natural and contaminated environments in the absence/presence of stressors different from antibiotics needs to be further studied (Finley et al., 2013; Marti et al., 2014; Zhang et al., 2018a).

This study aimed to determine the potential of environmental waterborne bacterial communities from urban and agricultural areas in the contaminated La Paz River basin to transfer genetic elements carrying multi-drug resistance into an *E. coli* lab strain as a recipient model. In addition, the effect of ZnSO_4_ and CuSO_4_ at sub-inhibitory concentrations as stressors during conjugation experiments was evaluated.

## Materials and methods

### Recipient strain and minimum inhibitory concentration determination

The strain *E. coli* CV601, characterized by the expression of the *gfp* gene (green fluorescent protein) and kanamycin (KAN) and rifampicin (RIF) resistance as selection markers (Heuer et al., 2002) was used as the recipient for conjugation experiments. The minimum inhibitory concentration (MIC) for sulfamethoxazole-trimethoprim (SMX/TMP) was tested prior to experiments using Etest^®^ (BioMérieux SA, Marcy l’Etoile, France) and was found to be 0.304–0.016 mg/L. *Escherichia coli* ATCC 25922 was used as a susceptibility control.

The MIC for the metal salts ZnSO_4_ × 7H_2_O (Merck, Darmstadt, Germany) and CuSO_4_ × 5H_2_O (Merck, Darmstadt, Germany) was determined by the agar dilution MIC determination method for metals (Aarestrup and Hasman, 2004) with some minor modifications. Briefly, Mueller-Hinton media was used to prepare series of two-fold dilutions of ZnSO_4_ × 7H_2_O (from 0.5 to 16 mM) and CuSO_4_ × 5H_2_O (from 0.5 to 32 mM). The pH of the media was adjusted to 5.5 and 7.0, respectively. Plates were inoculated with spots of 2 μL of bacterial suspensions adjusted to 0.5 McFarland standard. Plates were incubated for 48 h at 37°C, and the MIC concentration was established as the minimum concentration of metal salt where bacterial growth was not detectable (Wiegand et al., 2008). *Escherichia coli* ATCC 25922 was used as a control and for comparison purposes. MIC determination tests were conducted in triplicate. The MIC of the CV601 recipient strain was calculated as 4 mM for ZnSO_4_ × 7H_2_O and 16 mM for CuSO_4_ × 5H_2_O.

## Growth kinetics of the recipient strain in the presence of metals

Growth kinetic experiments were performed to test the effect of ZnSO_4_ × 7H_2_O and CuSO_4_ × 5H_2_O at two selected sub-inhibitory concentrations (0.5 and 1 mM) on the growth rate of the recipient strain. *Escherichia coli* CV601 recipient and *E. coli* ATCC 25922 (control) were cultured in Luria-Bertani (LB) broth (Sigma-Aldrich, St. Louis, MO, USA) for 3 h at 37°C, and the OD_600_ of each culture was adjusted to 1. Bacterial suspensions were added in aliquots of 20 μl to 96 well plates containing 180 μl of LB broth (negative control) or LB broth supplemented with ZnSO_4_ × 7H_2_O or CuSO_4_ × 5H_2_O at 0.5 and 1 mM. Plates were incubated overnight at 37°C, and the OD_600_ was measured using an automatic spectrophotometer (SpectraMax^®^ i3x, Molecular Devices, San Jose, CA, USA) every 30 mins. Growth kinetic experiments were performed twice with three replicates. The effect of metal addition on the growth rate of *E. coli* CV601 was taken into account for TF calculations by adjusting the recipient counts.

### Recipient and donor preparation prior conjugation experiments

*Escherichia coli* CV601 was grown in LB broth supplemented with KAN 50 mg/L at 37°C overnight. The culture was diluted 1:10 with LB broth without antibiotics and grown at 37°C for 2–3 additional hours. The recipient suspension was washed twice with PBS, pelleted, and finally resuspended in LB broth to an OD_600_ of 1–1.2 (≈1 × 10^9^ CFU/mL).

The collection of water samples from the highly contaminated La Paz River basin in Bolivia was carried out three times on different occasions and used as a source of donor communities for conjugation experiments. The first and second experiments were performed in July and August 2016, respectively, and the third experiment was performed in April 2018. The specific area and sampling sites are described elsewhere (Poma et al., 2016; Guzman-Otazo et al., 2019). An urban site located in the Choqueyapu River in La Paz city, where the river receives the discharge from hospitals and industries, and a downstream agricultural site where river water is used for crop irrigation were selected for water sampling. A total of three samples of water per site were collected to obtain 1 L of water, which was mixed, and subsequently an aliquot of 300 ml was taken and filtered through 0.45 μm pore size filters (Millipore Corporation, Bedford, MA, USA). The filters were cut, placed in tubes containing PBS and 5 mm glass beads (Supelco, Merck, Darmstadt, Germany), and vigorously vortexed to release the bacteria from the filters (donors). The donor suspension was decanted, washed twice with PBS, pelleted, and subsequently resuspended at an OD_600_ of 1–1.2. Water samples and donor suspensions were kept at 4°C at a maximum of 24 h before conjugation experiments.

### Conjugation assays

Conjugation assays were performed according to the protocol by Jutkina et al. (2016) with some minor modifications. Briefly, donor suspensions from each sampling point were mixed 1:1 with the recipient. Aliquots of 100 μL of donor-recipient suspensions were spread on a membrane of mixed cellulose esters (MCE) MF-Millipore™ with a 0.22 μm pore size and a diameter of 47 mm (Millipore Corporation, Bedford, MA, USA) and placed on LB media plates for mating. In the case of testing metal salts as stressors during conjugation experiments, donor-recipient mating filters were placed on LB media supplemented with ZnSO_4_ × 7H_2_O and CuSO_4_ × 5H_2_O at 0.5 and 1 mM. Control filters containing only the donor or the recipient suspensions on LB media plates were incubated and treated under the same conditions as the donor-recipient mating filters. After 3 h of incubation at 30°C, mating and control filters were transferred to tubes containing PBS and glass beads. The tubes were vortexed, and the contents serially diluted ten-fold. Plating of 100 μL of every dilution was performed on CHROMagar™ MH Orientation (CHROMagar, Paris, France), or standard Mueller Hinton Agar (MHA) (Sigma-Aldrich, St. Louis, MO, USA) supplemented with KAN (50 mg/L), RIF (50 mg/L), and the chosen selective antibiotic SMX/TMP (150/30 mg/L). This antibiotic combination was selected due to the high variety of donors and conjugative and transferable genetic elements characterized to carry sulfamethoxazole resistance genes (Jutkina et al., 2016). In addition, the mix of sulfamethoxazole/trimethoprim, commercially known as Cotrimoxazole, is frequently used to treat gastrointestinal and urinary tract infections in the Bolivian population, suggesting that the associated resistance genes are circulating in the population and surrounding environments (Duwig et al., 2014; Archundia et al., 2017, 2018). Control plates with recipients were plated onto MHA supplemented with KAN (50 mg/L) and RIF (50 mg/L), and control plates for donors onto CHROMagar™ Orientation or LB media. For counting and visualizing recipients and transconjugants, the plates were incubated at 37°C for 48 h. In the case of donors, plates were incubated at 30°C. Transfer frequencies (TFs) were calculated as the ratio between the number of transconjugants obtained from each specific condition and the number of recipients on the control plates.

Three independent experiments were performed. Every independent experiment included collecting water samples at the two sites and two mating replicates for each donor-recipient combination. Plating of bacterial dilutions was always performed in triplicate.

A second transconjugation experiment was performed using four selected transconjugants to verify that the acquired genetic elements were mobile. Transconjugation was performed as described above using four transconjugants with each type of IncN plasmid on an *E. coli* CV601 background as donors and *E. coli* HA4 (BW25113) (Alalam et al., 2020) as recipient. The mating efficiency was calculated as the ratio of transconjugant to donor colonies.

### Selection and confirmation of transconjugants

Transconjugants were identified based on growth in selective media and confirmed by *gfp* phenotype under UV light. For additional verification, selected transconjugants were tested by PCR to confirm the presence of the *gfp* gene construct as a marker of the recipient strain (Jutkina et al., 2016). Transconjugants were also tested by PCR for the presence of sulfamethoxazole resistance genes *sul1* and *sul2* to confirm the transfer of MGEs carrying resistance to the chosen selective antibiotic (Flach et al., 2015).

### Characterization of multi-drug resistant patterns obtained from conjugation experiments

A total of 150 randomly selected transconjugants, including 50 from each independent experiment and five from each of the ten sampling site-experimental condition combinations, were evaluated for phenotypic antibiotic resistance using the Kirby-Bauer Disk Disks (Oxoid/Thermo Fisher Scientific, Basingstoke, UK) Diffusion Susceptibility Test (Hudzicki, 2009). Disks for 13 antibiotics were tested: Ampicillin (10 μg), Tetracycline (30 μg), Ciprofloxacin (5 μg), Chloramphenicol (30 μg), Cefotaxime (5 μg), Meropenem (10 μg), Doripenem (10 μg), Ertapenem (10 μg, Imipenem (10 μg), Nalidixic Acid (30 μg), Gentamicin (10 μg), Streptomycin (10 μg), and Piperacillin-Tazobactam (110 μg). When possible, EUCAST guidelines^1^ were applied to interpret inhibition zones and categorize transconjugants as resistant, intermediate, or susceptible to the different antibiotics tested. CSLI guidelines^2^ were alternatively used when needed.

Every different combination of antibiotic susceptibility, resistance, or intermediate response to the 13 antibiotics tested was considered as a single MDRP. All transconjugants were classified as resistant to SMX/TMP as they were isolated from the conjugation assay using this antibiotic combination as the selection factor.

The multiple antibiotic resistance index (MAR) was calculated for each transconjugant as the ratio between the number of antibiotics to which the transconjugant is resistant and the total number of antibiotics tested. The Shannon diversity index (H’) for MDRPs was calculated using the Excel template developed by Klaus D. Goepel.^3^

### Evaluation of metal tolerance in selected transconjugants

Fifty randomly selected transconjugants representing both donor sites, all treatments, and all observed MDRPs were examined for tolerance to ZnSO_4_ × 7H_2_O and CuSO_4_ × 5H_2_O. In the case of MDRPs that were repeatedly found at different sites and treatments, more than one transconjugant was included in the analysis. In the case of unique MDRPs, only one transconjugant was available per site. Agar dilution MIC determination method for metals (Aarestrup and Hasman, 2004) was applied with some minor modifications as mentioned above. The acquired tolerance to metals was defined as an increase in the MIC value for the tested transconjugants compared to the MIC of the original recipient strain *E. coli* CV601. The strain of *E. coli* ATCC 25922 was also included as a control. Metal tolerance evaluation was conducted in triplicate.

### DNA extraction and whole-genome sequencing

DNA was extracted individually from 47 of the 50 selected transconjugants used in the metal tolerance test (representing the 28 different MDRPs identified in this study) and *E. coli* CV601 using the QIAamp Fast DNA Stool Mini Kit (Qiagen, Hilden, Germany). The DNA was eluted in Milli-Q water, and 50 ng of DNA were used for sequencing library preparation. Sequencing libraries were prepared using the TruSeq Nano kit (Illumina, San Diego, CA, USA) with a mean fragment length of 900 bp. Libraries were sequenced on the MiSeq platform using v3 chemistry, 2 × 300 bp, generating coverage of >100× for all strains.

### Sequencing data analysis

Sequencing data were processed using the BACTpipe assembly and annotation pipeline (v. 2.6.1) (Kirangwa et al., 2017). To find and subtract the acquired genes, the annotated genomes of the transconjugants were compared with the genome of the original recipient *E. coli* CV601. Contigs containing the acquired, transferred DNA were manually analyzed to identify antibiotic resistance genes (ARGs), other relevant genes (ORGs) conferring possible advantage to the host (biofilm and persister formation, survival under stress conditions, resistance to disinfectants, solvents or antimicrobial peptides and virulence), conjugation machinery (*tra* genes) and plasmid maintenance associated genes (toxin antitoxin systems), *etc.*, using ResFinder (Camacho et al., 2009; Bortolaia et al., 2020). The similarity of plasmid-like structures found in transconjugants with other previously reported plasmids was analyzed by NCBI BLAST (Altschul et al., 1990, 1997; Johnson et al., 2008). We further examined the content of the acquired genetic material in transconjugants by performing plasmid finding, plasmid sequence typing, antibiotic resistance gene finding, and virulence gene finding following the Bacterial Analysis Pipeline (Thomsen et al., 2016) in assembled genomes. Finally, metal and biocide resistance genes were searched for on the assembled genomes using DIAMOND BLASTx search (v. 0.9.25) (Buchfink et al., 2015) against the BacMet experimentally confirmed resistance gene database (v. 2.0) (Pal et al., 2014). The settings for BacMet analysis included “more-sensitive,” e-value > 10^–5^, amino acid identity > 90%, and the default alignment length cutoff.

### Statistical analysis

One- and two-way ANOVA in IBM SPSS Statistics (v. 22.0) with a significance level of 95% (*p* < 0.05) was used to test the effect of metal salts on the growth kinetics of *E. coli* CV601 and the effect of donor source and metal treatment on the number of transconjugants, TF, richness, diversity, and number of unique MDRPs per site when comparing multiple groups. *T*-test and Mann-Whitney *U* test were used when appropriate to compare variables between two groups. Non-metric multidimensional analysis NMDS was performed using R (v. 3.5.0). The “ecodist” (v. 2.0.1) R package was used to calculate Euclidean distances, and “vegan” R package was used to conduct NMDS and anosim analyses.

## Data availability

All of the genomes determined in this study are available at NCBI under BioProject no. PRJEB33390. The sequences and strain features are deposited in the European Nucleotide Archive under the accession number PRJEB33390.

## Results

### Culturable bacterial donor communities in the La Paz River basin

Water samples from the two sites at the La Paz River basin were plated on CHROMagar orientation media to characterize the culturable fraction of donor communities. Bacteria culturable on CHROMagar were abundant at both the urban (9.6 × 10^5^CFU/ml on average) and agricultural (4.3 × 10^5^ CFU/ml on average) sites (Supplementary Figure 1). No statistically significant difference was found between bacterial counts from the two sites. The bacterial genera were classified solely based on the appearance of observed colonies^4^ and included: *E. coli*, *Enterococcus*, *Klebsiella, Enterobacter, Serratia*, *Staphylococcus aureus*, *and Pseudomonas aeruginosa*, with no apparent differences in composition between sampling sites (Supplementary Figure 1). It is important to note that donors were prepared directly from water filters and without any prior culture step, so donor communities from the La Paz River basin included culturable and non-culturable bacteria for mating experiments.

### Bacterial communities from urban and agricultural areas in the La Paz River basin have the potential to transfer antibiotic resistance to *Escherichia coli*

**FIGURE 1:**
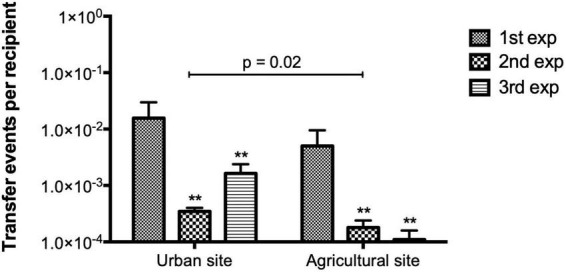
Transfer frequencies (TFs) obtained from conjugation experiments in LB media using aquatic donor communities from urban and agricultural areas of the La Paz River basin and *E. coli* CV601 (recipient). Bars represent mean values obtained from three independent conjugation experiments. Error bars represent the standard deviation of the mean from the two mating replicates included in each independent experiment. A higher number of transfer events per recipient was obtained using bacterial donors from the urban site (Mann-Whitney *U* Test, *p* = 0.02). For both sampling sites, TFs varied significantly between independent experiments. Asterisks (**) show the significantly between difference from the first independent experiment (1st exp) by Brown-Forsythe ANOVA *p* = 0.03, *post hoc* Tukey test. There was no significant difference in TF between the second and third independent experiments.

Conjugation experiments in LB media using bacterial communities from the two sites at the La Paz River basin as donors and *E. coli* CV601 as the recipient were performed. The number of transconjugants and TFs were calculated from three independent experiments. When combining all three experiments, significantly higher numbers of SMX-TMP resistant transconjugants were obtained using donor communities from the urban area (7 × 10^4^ CFUs) compared to the agricultural area (1.5 × 10^4^ CFUs) (Mann-Whitney *U* = 21, *p* = 0.003 two-tailed). Significantly higher TFs were obtained using donor communities from the urban site compared to the agricultural area (Mann-Whitney *U* = 32.5, *p* = 0.02 two-tailed; Figure 1). Hence, the urban site might contain higher levels of suitable donors. Additionally, a statistically significant difference in TF between independent experiments was observed in both sampling sites (Brown-Forsythe ANOVA F = 5.97, *p* = 0.03; Figure 1).

### Transfer of antibiotic resistance from waterborne bacterial communities to *Escherichia coli* in the presence of stressors ZnSO_4_ and CuSO_4_

Donors from the two sites along the La Paz River basin were also used for conjugation experiments in LB media supplemented with sub-MIC levels of ZnSO_4_ × 7H_2_O and CuSO_4_ × 5H_2_O to test if stressors might influence the transfer of resistance determinants. The selected concentrations were 0.5 and 1 mM for both metals, since the objective was to exert stress on donors and recipients but not kill them during mating experiments. For this reason, the selected concentrations were under the MIC but higher than the expected concentrations in watersheds. Information about the metal content in the La Paz River basin is scarce.

We monitored the growth of the recipient *E. coli* CV601 and control *E. coli* ATCC 25922 in the presence of ZnSO_4_ × 7H_2_O and CuSO_4_ × 5H_2_O at 0.5 and 1 mM overnight. Since conjugation experiments were performed with incubation of 3 h, this time point was selected for statistical analysis and further interpretation. Growth was not significantly affected in the presence of 0.5 mM and 1 mM ZnSO_4_ or by 0.5 mM CuSO_4_, while CuSO_4_ at 1 mM caused a reduction of 29% in the bacterial growth of the recipient (One-Way ANOVA *p* < 0.001) at 3 h of incubation at 37°C (Supplementary Figure 2). This effect was continuously observed up to 6 h after incubation (One-Way ANOVA *p* < 0.001; Supplementary Figure 2). Comparable growth kinetic results were observed for the control strain of *E. coli* ATCC 25922. The reduction in growth produced by CuSO_4_ at 1 mM was included in the TF calculations.

**FIGURE 2:**
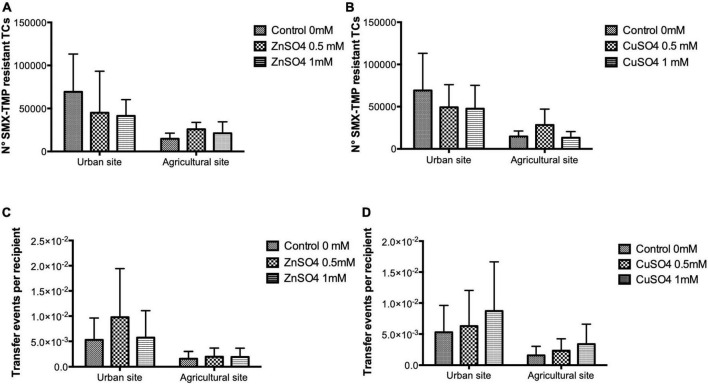
The number of transconjugants and transfer frequencies (TFs) obtained from conjugation experiments in LB media supplemented with ZnSO_4_ and CuSO_4_ at 0.5 and 1 mM. Conjugation experiments used waterborne bacterial donor communities from two La Paz River basin sites and *E. coli* CV601 as the recipient strain. The bars represent mean values of the number of SMX-TMP resistant transconjugants **(A,B)** and transfer events per recipient **(C,D)** in the presence of ZnSO_4_ and CuSO_4_. Data were obtained from three independent conjugation experiments. The error bars represent the standard deviation of the mean. For both sampling sites, the number of SMX-TMP-resistant transconjugants and TFs did not differ significantly between the experiments performed in LB media and LB media supplemented with metals.

Adding ZnSO_4_ and CuSO_4_ at 0.5 and 1 mM to conjugation experiments did not cause any significant change in the number of SMX-TMP resistant transconjugants and TFs compared to conjugation experiments in LB media without the addition of metals for both sampling sites (Figure 2). Hence, the sub-MIC metal levels evaluated in this study did not significantly affect the TF of antibiotic resistance.

### Multi-drug resistance profiles were acquired from conjugation experiments using waterborne bacterial donors and metal salts as stressors

**FIGURE 3:**
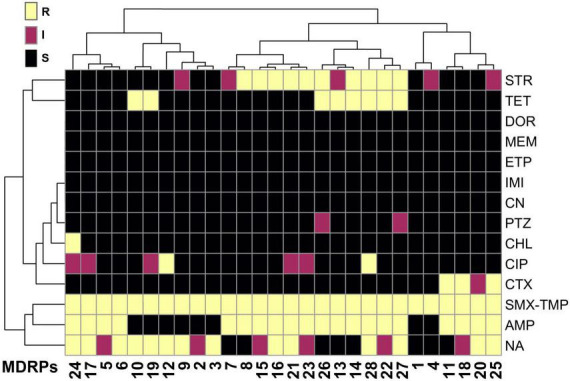
Heatmap of the phenotypic characterization of the main multi-drug resistance profiles (MDRPs) identified by the Kirby-Bauer Disk Diffusion Susceptibility Test in randomly selected transconjugants. A total of 28 different phenotypic MDRPs were transferred from bacterial communities from the two sites in the La Paz River basin to *E. coli* CV601 recipients. A single profile was defined as the unique combination of susceptibility, intermediate resistance, or resistance to the 13 antibiotics included in the analysis. Color code: black = susceptible (S), magenta = intermediate (I), and yellow = resistant (R). Antibiotics: AMP, ampicillin; TET, tetracycline; CIP, ciprofloxacin; CHL, chloramphenicol; CTX, cefotaxime; MEM, meropenem; DOR, doripenem; ETP, ertapenem; IMI, imipenem; NA, nalidixic acid; CN, gentamicin; STR, streptomycin; PTZ, piperacillin-tazobactam; SMX/TMP, sulfamethoxazole/trimethoprim. All MDRPs include resistance to SMX/TMP because all transconjugants were selected from conjugation experiments using plates supplemented with this specific combination of antibiotics. Correlation was used as the clustering method. The pheatmap R program was used to generate the heatmap.

Even if the addition of stressors did not affect the total number of transconjugants or TFs, it might cause selective pressure on the transferred resistome. To determine the transposable genetic elements of the two sampling sites with and without stressors, 150 randomly selected transconjugants from experiments in the absence and presence of metals were analyzed using the Kirby-Bauer Disk Diffusion Susceptibility Test for 13 different antibiotics. Considering that SMX-TMP plates were used to select transconjugants, resistance to this combination of antibiotics was found in every MDRP (Supplementary Table 1). After classification of each transconjugant as susceptible, intermediate, or resistant to each antibiotic tested, a total of 28 different phenotypic MDRPs among the 150 isolates tested were identified. Each profile represented a specific combination of susceptibility, intermediate resistance, and resistance to the group of antibiotics tested (Figure 3 and Supplementary Table 1). As shown in the clustering analysis in Figure 3, MDRPs obtained using SMX-TMP resistance as the selection marker for transconjugants were found to commonly include resistance to ampicillin, nalidixic acid, and streptomycin, and less frequent to tetracycline and cefotaxime. Resistance to ciprofloxacin and chloramphenicol was rarely found in the MDRPs obtained in this study. Intermediate resistance to piperacillin-tazobactam was identified in two transconjugants. Resistance to gentamicin and carbapenems was not detected in any MDRP (Figure 3 and Supplementary Table 1).

**FIGURE 4:**
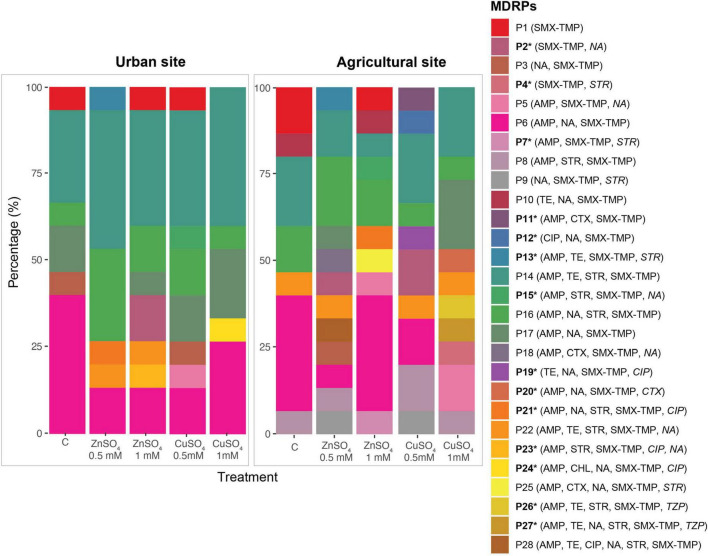
Distribution and abundance of MDRPs obtained from conjugation experiments in the absence (C) and presence of ZnSO_4_ and CuSO_4_ at 0.5 and 1 mM. Urban and agricultural areas in the La Paz River basin were used as the source of bacterial donor communities. A total of 28 MDRPs (P1–P28) were identified in 150 randomly selected transconjugants analyzed by the Kirby-Bauer Disk Diffusion Susceptibility Test to 13 antibiotics. Each identified profile was labeled with a distinctive color followed by the antibiotic resistance profile. Antibiotics whose transconjugants exhibited intermediate resistance are written in italics. Unique MDRPs per site are marked with a star (*), and they were only found with the addition of metals in conjugation experiments.

The Shannon index of diversity (H’) and richness (R) of MDRPs obtained from conjugation experiments with and without the addition of ZnSO_4_ and CuSO_4_ were calculated. When conjugation experiments were performed in the absence of metals, the diversity and richness of MDRPs did not vary significantly between the two sampling sites since a richness of six and seven different MDRPs was identified, respectively (Table 1). Some of these profiles like P1 (STX/TMP), P6 (STX/TMP, AMP, and NA), P14 (STX/TMP, AMP, TET, and STR), and P16 (STX/TMP, AMP, NA, and STR) were found from both sampling sites (Table 1, Supplementary Table 1, and Figure 4). Unique profiles per site were defined as profiles detected only once among all transconjugants tested per sampling site. When metals were added to the conjugation experiments and data from both metal treatments was analyzed as a group, a tendency of variation in the richness of MDRPs between sampling sites was observed (Mann-Whitney *U* = 40 *p* = 0.052; Table 1). In addition, a statistically significant difference between the two sites was observed for the percentage of unique MDRPs per site in the presence of metals (Mann-Whitney *U* = 36.5 *p* = 0.026; Table 1). Thus, with the addition of metals to the conjugation media, the La Paz River donor bacteria from the agricultural site transferred more diverse MDRPs than bacterial donors from the urban point (Table 1 and Figure 4). Notwithstanding that, the TFs obtained from the agricultural area were markedly lower compared to the TFs obtained from the urban site (Figure 2).

**TABLE 1:** Number of multi-drug resistance profiles (MDRPs) obtained from conjugation experiments in the absence and presence of ZnSO_4_ and CuSO_4_ at two different concentrations.

	Transconjugants							AR profiles		
		
Treatment	1A (%)^a^	2A (%)^a^	3A (%)^a^	4A (%)^a^	5A (%)^a^	6A (%)^a^	MAR^b^	R^c^ (U%)^d^	H’^e^	H’^e^ (R)^c^
**Urban site**										
Control 0 mM	1 (7)	1 (7)	8 (53)	5 (33)	0 (0)	0 (0)	0.22	6 (0)	1.53	1.53 (6)
ZnSO_4_ 0.5 mM	0 (0)	0 (0)	3 (20)	12 (80)	0 (0)	0 (0)	0.27	6 (33)	1.53	1.89 (10)
ZnSO_4_ 1 mM	3 (20)	0 (0)	4 (27)	8 (53)	0 (0)	0 (0)	0.22	8 (25)	1.89	
CuSO_4_ 0.5 mM	1 (7)	2 (13)	5 (33)	7 (47)	0 (0)	0 (0)	0.23	8 (25)	1.89	1.79 (9)
CuSO_4_ 1 mM	0 (0)	0 (0)	7 (47)	8 (53)	0 (0)	0 (0)	0.25	5 (20)	1.40	
**Agricultural site**										
Control 0 mM	2 (13)	0 (0)	7 (47)	6 (40)	0 (0)	0 (0)	0.22	7 (0)	1.77	1.77 (7)
ZnSO_4_ 0.5 mM	1 (7)	2 (13)	5 (33)	6 (40)	0 (0)	1 (7)	0.24	12 (33)	2.40	2.67 (19)
ZnSO_4_ 1 mM	1 (7)	2 (13)	7 (47)	5 (33)	0 (0)	0 (0)	0.22	10 (50)	2.08	
CuSO_4_ 0.5 mM	2 (13)	1 (7)	7 (47)	5 (33)	0 (0)	0 (0)	0.21	10 (30)	2.21	2.50 (15)
CuSO_4_ 1 mM	1 (7)	0 (0)	7 (47)	6 (40)	1 (7)	0 (0)	0.24	10 (40)	2.18	

^a^Number and percentage of transconjugants with resistance to 1, 2, 3, 4, 5, and 6 different antibiotics.

^b^Multiple antibiotic resistance index (MAR) calculated as the ratio between the number of antibiotics to which the transconjugant is resistant and the total number of antibiotics tested.

^c^Richness or number of different antibiotic susceptibility profiles observed.

^d^Percentage of unique profiles observed in transconjugants obtained from a specific treatment and per sampling point. ^e^Shannon Diversity Index (H’).

The analysis was also performed per individual sampling point for diversity, richness, and unique MDRPs in the absence and presence of metals. When the urban site was used as a donor source, no significant differences were observed in the diversity, richness, and unique MDRPs between conjugation experiments in the absence and presence of metal treatment (Table 1). However, when the agricultural site was used as the source of donors, a significantly higher diversity (*t*-test, *p* = 0.028), richness (Mann-Whitney *U* = 3 *P* = 0.021), and percentage of unique MDRPs (Mann-Whitney *U* = 3, *p* = 0.023) was obtained from conjugation experiments in the presence of metals compared to LB only (Table 1).

The multiple antibiotic resistance index (MAR) varied slightly between sampling sites and treatments, with a small range of 0.22–0.27 (Table 1). Only 7% of the transconjugants acquired resistance to more than five antibiotics, and this was only found in transconjugants from the agricultural site obtained from conjugation experiments in the presence of 0.5 M ZnSO_4_ (Table 1).

The number of resistant transconjugants to each of the 13 antibiotics tested, and the number of transconjugants carrying each of the 28 different MDRPs were compared between sampling sites and metal treatments. In the absence of metal treatment, no significant differences were observed between the sampling sites. When metals (ZnSO_4_ and CuSO_4_) were added to the conjugation media, transconjugants with intermediate resistance to piperacillin-tazobactam were only found in the agricultural area. Transconjugants expressing intermediate resistance to streptomycin (STR) were significantly enriched on the agricultural site compared to the urban site (Mann-Whitney *U* = 42, *p* = 0.028). Transconjugants carrying the STX/TMP, AMP, STR resistance profile (P8) were significantly more often present in the agricultural area (Mann-Whitney *U* = 48, *p* = 0.032), while P14 (STX/TMP, AMP, TET, and STR) was significantly enriched in the urban site (Mann-Whitney *U* = 35.5, *p* = 0.028; Figure 4). Finally, the number of transconjugants resistant to streptomycin was significantly higher in conjugation experiments with metal treatment (Mann Whitney *U* = 5, *p* = 0.046), and P6 (STX/TMP, AMP, and NA) was significantly enriched in experiments in the absence of metals (Mann Whitney *U* = 30, *p* = 0.022; Figure 4).

## The origin of the sample and the presence of metals determined the acquisition of specific antibiotic resistance genes

**FIGURE 5:**
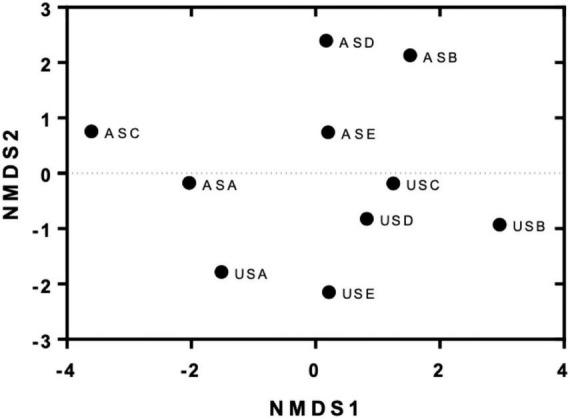
Non-metric multidimensional scaling (NMDS) of obtained transconjugants from conjugation experiments in the absence and presence of metals. The results show the ordination of different groups of transconjugants in two dimensions according to the similarities in their MDRPs. US: transconjugants were obtained using the urban site as the donor source. AS: Transconjugants were obtained using the agricultural site as the donor source. Treatments: A = without the addition of metals, B and C = 0.5 and 1 mM ZnSO4, respectively, D and E = 0.5 and 1 mM CuSO4, respectively.

Similarities between transconjugants obtained from experiments in the absence and presence of metals were evaluated according to the occurrence and abundance of specific MDRPs using a non-metric multidimensional scaling (NMDS) analysis. The results shown in Figure 5 showed that transconjugants obtained from the urban site (US) and the agricultural site (AS) clustered separately in the space, meaning that transconjugants obtained from these two places acquired different combinations and abundance of MDRPs (Figure 5). Moreover, transconjugants obtained from conjugation experiments in the presence of metals (B: LB + 0.5 mM ZnSO_4_, C: LB + 1 mM ZnSO_4_, D: LB + 0.5 mM CuSO_4_, and E: LB + 1 mM CuSO_4_) (Supplementary Table 2) clustered together and separately from transconjugants obtained in the absence of metals (A: LB) (Figure 5). An exception was observed for transconjugants obtained from the agricultural site and 1 mM ZnSO_4_ (ASC). This group of transconjugants was located far from the other groups of transconjugants obtained in the presence of metals (Figure 5).

### Tolerance to ZnSO_4_ and CuSO_4_ in transconjugants from conjugation experiments in the absence and presence of metals

Since the results suggested that adding ZnSO_4_ and CuSO_4_ might influence the transfer/uptake of different pools of MGEs compared to conjugation experiments in the absence of metals, we next determined if the transconjugants had obtained increased metal tolerance. A subgroup of 50 transconjugants representing all MDRP types obtained from conjugation experiments in LB media with and without the addition of ZnSO_4_ and CuSO_4_ was evaluated for increased tolerance to both metal salts compared to those obtained to the recipient strain of *E. coli* CV601. All tested transconjugants presented the same MIC values as the recipient: 4 mM for ZnSO_4_ and 16 mM for CuSO_4_. An increased metal tolerance was not obtained for the presence of the sub-MIC metal levels used in this study.

### Genomic profiling of antibiotic resistance genes, other relevant genes, and plasmids among representative multi-drug resistant patterns

**FIGURE 6:**
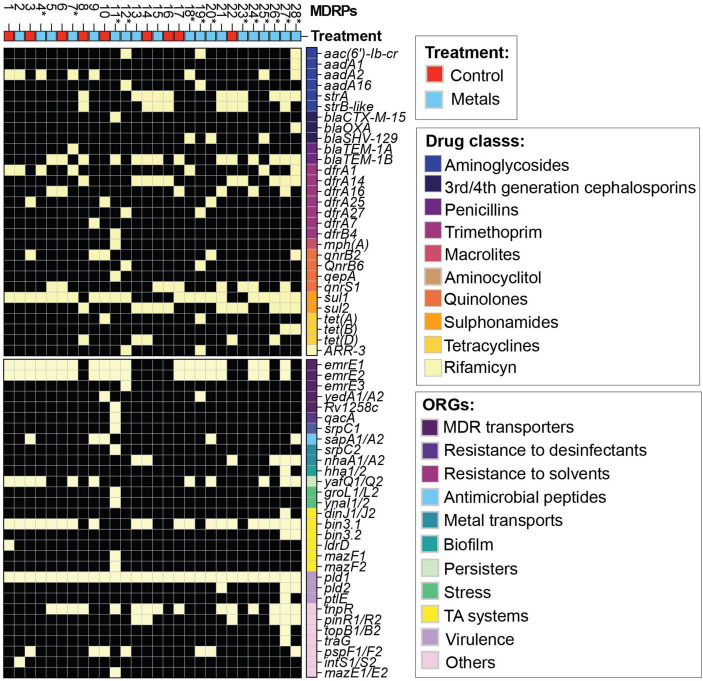
Genomic characterization of representative transconjugants with unique MDRP. Heatmap based on the presence (yellow)/absence (black) of ARGs annotated using the ResFinder database (Bortolaia et al., 2020). ARGs were classified according to the drug class they belong to. Other relevant genes (ORGs) were labeled based on their biological function. Unique MDRPs are marked with (*).

The antibiotic disc diffusion test and the NMDS analysis indicated that site-specific MGEs might be present at the two sites analyzed in the present study. To determine the transferred MGEs, we performed WGS of 47 selected transconjugants, representing all MDRPs and the recipient strain of *E. coli* CV601 (Supplementary Table 2). The assembled and annotated CV601 genome was subtracted from the transconjugant genomes to identify the acquired MGEs. To get a more comprehensive characterization of the ARGs, the ResFinder (Bortolaia et al., 2020) database was used to annotate and identify the resistance genes within the transconjugants’ genomes. In general, several ARGs acquired by this group of transconjugants were correlated with the observed phenotypic resistance (Figures 3, 4 and Supplementary Table 1). However, some exceptions were observed. For example, P2 and P18 showed intermediate resistance to nalidixic acid (NA), but no apparent plasmid-borne quinolone resistance genes or mutations in the nucleotide sequence of DNA gyrase subunit B (*gyrB*) were identified. Conversely, P1-P2 and P8 profiles displayed susceptibility to streptomycin despite the presence of *aadA2/strAB* genes (Figures 4, 6 and Supplementary Table 1).

Unique MDRPs P11, P18, P20, P25, and P28 carried extended-spectrum B-lactamases (ESBLs) such as *bla*_CTX–M–15_, *bla*_SHV–129_, and *bla*_OXA–9_ were only obtained from conjugation experiments in the presence of metals and using the agricultural site as the source of donors (Figure 6). After ARGs identification, we identified other relevant genes (ORGs) in the MGEs acquired by transconjugants that might favor the host. We found genes encoding multi-drug transporters (*yedA, Rv1258c*), genes conferring resistance to other agents such as disinfectants (*qacA, emrE1*, or *qac*Δ*E1*), solvents (*srpC1*) or antimicrobial peptides (*sapA*), genes encoding metal transporters (*nhaA, srpC2*), genes involved in biofilm and persister formation (*hha, yafQ*) as well as survival in stress conditions (*groL, ynaI*), toxin anti-toxin system genes (*dinJ, ldrD*, *mazE*, and *mazF*) and virulence genes (*pld, ptlE*) among others. The transconjugants also carried higher varieties of ORGs involved in resistance to disinfectants and solvents, metal transporters, biofilm formation, survival in stress conditions, and virulence were mainly obtained from conjugation experiments in the presence of metals (Figure 6). Using VirulenceFinder (Joensen et al., 2014; Malberg Tetzschner et al., 2020) only two virulence genes, glutamate decarboxylase (*gad)* and increased serum survival (*iss)*, were found equally present in the recipient strain and all transconjugants. In addition, we were able to identify that the transconjugants also acquired the class 1 integron-integrase gene, *intl1*, which is an a proxy for anthropogenic pollution (Gillings et al., 2015). This gene was found in all transconjugants (Supplementary Table 2).

By comparing the genomes size of the transconjugants against the recipient *E. coli* CV601 genomes, we were able to get information on the size of genomic DNA transferred from waterborne bacterial communities to *E. coli* CV601. The size of the transferred genomic DNA ranged from 38 Mbp (ASE-3-8) to 164 Mbp (ASE-3-3). No significant difference was observed in the approximate number of base pairs acquired by transconjugants from both sampling sites and in the absence or presence of metals (Supplementary Table 1). The transferred genomic DNA was most likely plasmids and profiling of the plasmid multi-locus sequence types (pMLSTs) and plasmid incompatibility groups (Inc group) of the acquired genome identified that all transconjugants acquired at least one plasmid from the IncN group (IncN, Inc2, and IncN3). pMLST analysis showed that the IncN plasmids belonged to ST5, ST6 or an unknown type. The resistance gene *sul1* was carried mainly by IncN2 and IncN3 plasmids, while *sul2* was always located in IncN plasmids (Supplementary Table 2). Further characterization of the methylation-related genes that play a role in the bacterial restriction-modification system (RM) and plasmid transfer efficiency identified five different methylation-associated genes that encode MTases such as Dcm (*dcm*), HsdM (*hsdM*) genes, and *ECO*RII (*ecoRIIM*), and Reases as HsdR (*hsdR*), and *klcA2*. IncN plasmids carried *ecoRIIM*, *dcm*, and some carried *klcA2*, IncN2 plasmids carried *hsdM/hsdR*, and IncN3 plasmids carried *klcA2* (Supplementary Table 1).

**FIGURE 7:**
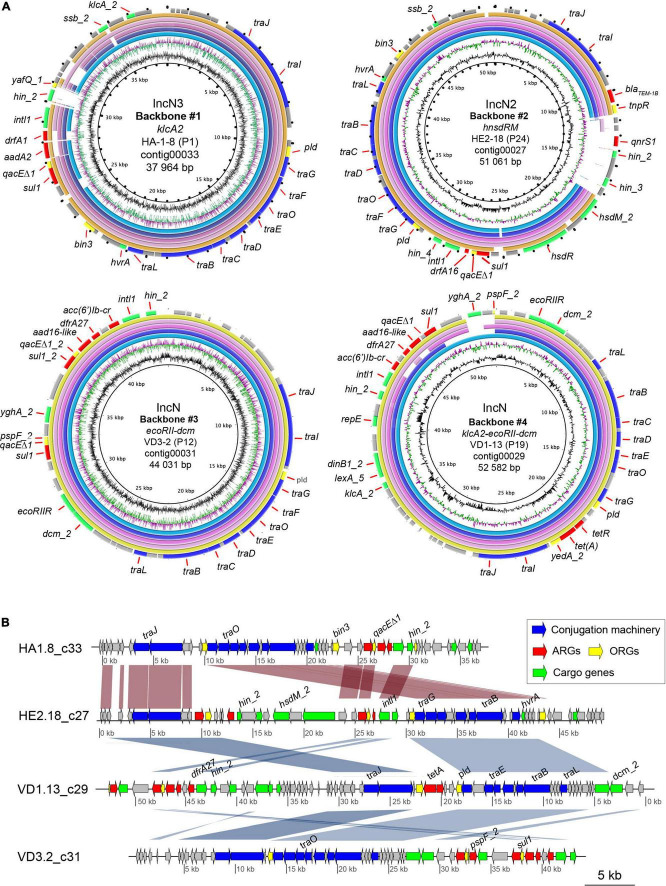
Comparison between the IncN plasmid-like backbones repeatedly identified in transconjugants from river water using blastn. **(A)** The top five plasmid genomes with the highest percentage of identity (>70%) and query cover were included in the comparison. A plasmid-like backbone was used as the reference sequence (outer ring). The genes present in the reference genome are shown as black dots in the rings that represent the five genomes. The color code for the annotations is listed at the bottom of the figure. The two inner rings depict GC content in black and GC Skew- in purple, and GC Skew + in green. The figures were generated using BRIG36 (v0.95, http://brig.sourceforge.net/). #1: *K. pneumoniae* isolate JIE137 plasmid pJIE137 (EF219134.3) (light blue); *E. coli* I23 plasmid pEcI23 (MH713706.1) (purple); *K. pneumoniae* N11 plasmid pKpnN11 (MH782635.1) (magenta); *E. hormaechei* subsp. hormaechei strain 34983 plasmid p34983-59.134kb (CP010378.1) (pink); *E. coli* strain M17224 plasmid p17511_70 (MN583554.1) (yellow). Backbone #2: *E. coli* strain Ecol_448 plasmid pEC448_OXA163 (CP015078.1) (light blue); *E. coli* strain M17224 plasmid p17511_70 (MN583554.1) (purple); *K. pneumoniae* N11 plasmid pKpnN11 (MH782635.1) (magenta); *E. coli* I23 plasmid pEcI23 (MH713706.1) (pink); *C. farmeri* strain CCRI-24236 plasmid pCCRI24236-2 (CP081316.1) (yellow). Backbone #3: *K. pneumoniae* strain D17KP0013 plasmid pD17KP0013-2 (CP052348.1) (light blue); *Shigella flexneri* strain AR-0423 plasmid pAR-0423-1 (CP044159.1) (purple); *Enterobacter hormaechei* (*E. hormaechei*) strain Eho-E3 plasmid pEclE3-3 (CP049024.1) (magenta); *E. hormaechei* strain Eho-E4 plasmid pEclE4-3 (CP048699.1) (pink); *E. coli* strain RHBSTW-00822 plasmid pRHBSTW-00822_3 (CP056317.1) (yellow). Backbone #4: *K. pneumoniae* strain D17KP0013 plasmid pD17KP0013-2 (CP052348.1) (light blue); *K. pneumoniae* strain D16KP0144 plasmid pD16KP0144-3 (CP052359.1) (purple); *Salmonella enterica* subsp. enterica serovar Typhimurium strain STM3224 plasmid pUY_STM62 (MN241904.1) (magenta); *E. coli* strain Ec19397 plasmid pEc19397-131, complete sequence (MG878866.1) (pink); *E. hormaechei* strain EH_316 plasmid pEH_316-3 (CP078058.1) (yellow). **(B)** Comparison of the plasmid-like backbones of IncN plasmids. The MAUVE-based progressive multi-alignment shows the similarity between the genetic structure of the different IncN plasmid-like obtained during conjugation experiments. VD1.13_c29 nucleotide sequence was reversed to improve visualization of the comparison. The scale bar represents the sequence length. Protein annotations colored by putative functions are shown as arrows for each contig. BLAST similarity values greater than 95% between contigs are shown in red if they are oriented in the same direction and blue if they are in the reverse direction.

Additionally, sequence analysis using the BacMet database (Pal et al., 2014) revealed the presence of 131 biocide- and metal- resistance genes in the recipient *E. coli* CV601 (Supplementary Table 3). Compared to the recipient, only two extra biocide resistance genes (*qacE* and *qacEΔ1*) conferring resistance to quaternary ammonium compounds (QACs) were found in more than half of sequenced transconjugants, which were also characterized to carry the sulfonamide resistance gene *sul1* (Supplementary Tables 1, 3). Alignment and sequence analysis revealed that *qacEΔ1* was located adjacent to *sul1*, and in the same contig and plasmid as the class 1 integron integrase intI1 (Figures 7A,B).

Analysis of the genetic context of ARGs, ORGs and class 1 integron revealed that both were often found together in the same contigs within transconjugant isolates, indicating co-transfer on the same MGE or plasmid (Figures 7A,B). Contigs were found to be up to 55 kb and contained transferred genes. In addition, we found that several of the transconjugants with similar or identical MDRPs contained the same acquired genes with conserved gene order on identical contigs. Manual inspection of all contigs containing acquired genetic material verified that only four different and complete plasmid backbones with variations mainly in MGEs carrying ARGs were repeatedly identified among transconjugants from the river water microbial community. Since all four backbones were identified in transconjugants from urban and agricultural sites and from experiments in the absence and presence of metals, they were further studied (Figures 7A,B). The four plasmid backbones were classified according to the RSGs and Inc group. Backbone #1 was characterized to only carry the anti-restriction protein gene *klcA2* (IncN3), backbone #2 carried a combination of restriction-methylase genes *hsdR-hsdM* (IncN2), backbone #3 harbored a different combination of restriction-methylase genes *ECORII-dcm* (IncN) *and* finally, backbone #4 carried three different restriction system genes *klcA2-ECORII-dcm* (IncN) (Figure 7A).

In order to determine from the short-read assembles whether the acquired DNA was associated with AMR plasmids, the extra contigs were subjected to NCBI BLAST. The results showed that the four plasmid-like backbones were highly similar (85–95%) and covered a large proportion of the nucleotide sequences (75–90%) of AMR plasmids’ sequences found in the NCBI database and belonged to several bacterial pathogens such as *Klebsiella pneumoniae* (*K. pneumoniae*), *Enterobacter*, *Citrobacter*, and *Shigella*. For instance, the plasmid-like backbone #1 was found to be 93% similar to AMR IncN plasmid found in a clinical strain of *K. pneumoniae* JIE137 (EF219134.3) carrying *bla*_CTX–M–62_ isolated in Australia as well as in a clinical isolate of *E. coli* (EF219134.3) (Zong et al., 2008). Also, the plasmid-like backbone #1 was 95% similar to a 53.129 kb (77% coverage) MDR plasmid found in *Enterobacter hormaechei* (*E. hormaechei*) subsp. Steigerwaltti (CP010382.1) isolated from patients at different health care institutions in New York, USA. Although this plasmid was reported to carry several *bla* genes (*bla*_KPC–4_, *bla*_TEM–1A_, and *bla*_OXA–1_), they were not present in our transconjugants (Chavda et al., 2016). The comparison of the plasmid-like backbone #2 yielded MDR plasmids with an identity of almost 100% and coverage of 88% found in *E. coli* (pEC448_OXA163: CP015078.1) isolated from Argentina carrying an OXA-163 that was not acquired by our recipient strain. Similarly to plasmid-like backbone #1, plasmid-like backbone #2 was highly similar to a plasmid in *E. coli* p17511_70 (MN583554), which also carried a *bla*_OXA–48_ gene. Other hits for backbone #2 included *Citrobacter farmer* (*C. farmer*), a serially isolated KPC-2 producer from a single patient. The contig corresponding to the plasmid-like backbone #3 shared the highest coverage and pairwise nucleotide identity with other reported plasmids such as pD17KP0013-2 in *K. pneumoniae* (CP052348.1), pAR-04232-1 in *S. flexneri* AR-0423 (CP044159.1), pEclE3-3 in *E. hormaechei* strain Eho-E3 (CP049024.1). Although *K. pneumoniae* and *S. flexneri* were clinical isolates, *E. hormaechei* was recovered from sewage water samples from Ontario, Canada (Kohler et al., 2020). Lastly, plasmid-like backbone #3 and plasmid-like backbone #4 shared a highly similar DNA sequence with related plasmids from diverse bacterial species, and both plasmid-like backbones #3 and 4 are identical to pD17KP0013-2 found in *K. pneumoniae* and *E. hormaechei* strain Eho-E3 with the exception that plasmid-like backbone #4 is ∼12 kb larger than backbone #3 (Figure 7A).

A detailed comparison of the four plasmid-like backbones is shown in Figure 7B, where all backbones presented conserved regions containing mainly plasmid maintenance genes. All ARG positive contigs contained T4SS genes indicating the presence of the complete Tra-operon, including conjugation-associated genes *traD* and *traI*. Variable regions containing different combinations of ARGs, ORGs, metabolic and hypothetical genes were also identified in the four putative plasmid structures. Differences in the variable regions containing ARGs of the four plasmid-like structures determined the observed differences in phenotype and genotype of analyzed transconjugants. We confirmed that all identified backbones were mobile by a second transconjugation using four selected transconjugants representing #1–4 as donors and *E. coli* HA4 as the recipient.

## Discussion

The potential of waterborne bacteria from the contaminated La Paz River basin to transfer antibiotic resistance determinants to *E. coli* was evaluated. Two different sampling sites in the river were used as a donor source, one urban site located after the city center of La Paz and close to several hospitals, and another rural agricultural site located downstream in the river where water is used for irrigation of crops. After 3 h of mating experiments on solid media without adding stressors, SMX/TMP resistant transconjugants were retrieved at high frequencies from both sites in the La Paz River. Donors from the urban site generated more than three times higher TFs than the agricultural site (5.3 × 10^–3^ and 1.6 × 10^–3^ transfer events per recipient, respectively). Previous studies using waterborne donors and *E. coli* CV601 as the recipient reported transconjugants at frequencies of 1 × 10^–4^ transfer events per recipient in a lake in India (Flach et al., 2015) and 2 × 10^–6^ and 3 × 10^–5^ after 3- and 16-h mating experiments, respectively, using bacteria from a Swedish sewage treatment plant (Jutkina et al., 2016). Compared to these studies, bacterial donors from the La Paz River transferred SMX-associated MGEs to *E. coli* at very high frequencies after only 3 h of mating, suggesting that higher levels of bacterial contamination, particularly in the urban site, were associated with higher TFs of antibiotic resistance. Supporting these results, we reported in a previous study high bacterial loads of diarrheagenic *E. coli* (DEC), *Salmonella enterica*, *K. pneumoniae*, and *Shigella* spp., at different points in the La Paz River basin, including the urban and agricultural sites used in the present study. High water conductivity and the highest number of total enterobacteria (7 × 10^6^ *gapA* gene copies per 100 ml of river water) were observed at the urban site, confirming the high level of anthropogenic fecal contamination in the river and a high number of suitable donors for HGT experiments (Guzman-Otazo et al., 2019).

In the present study, the addition of metal salts such as ZnSO_4_ and CuSO_4_ at 0.5 and 1 mM in mating experiments did not cause a significant effect on the number of SMX-TMP resistant transconjugants and TFs. Previous studies on the effect of metals on conjugative transfer rates have shown contradictory results that are highly dependent on the type of metal and concentration tested. Suzuki et al. (2012) reported that vanadium at 0.5 and 1 mM significantly increased the conjugative transfer rate of oxytetracycline resistance plasmids from *Photobacterium damselae* strain 04Ya311 to *E. coli* JM109. However, other metals, such as zinc and copper, up to 0.5 mM caused a decrease in the conjugation rate of this study model. In another study, Zhang et al. (2018b) reported that very low concentrations of copper (0.005, 0.01, and 0.05 mg/L), silver (0.01 and 0.02 mg/L), and chromium (0.1 mg/L) but not zinc, significantly increased the conjugation frequency of a multi-drug resistance plasmid between two *E. coli* isolates in water conjugation experiments. It is important to note that the positive copper concentrations used by the authors were more than 100 times lower than the concentrations tested in our study. This might suggest that metals like copper are more likely to exert a promoting effect on conjugation rate at low concentrations. Metal nanoparticles have also been studied for their effect on bacterial conjugation. Zinc nanoparticles up to 10 mg/L increased the conjugative transfer of the resistance plasmid RP4 between *E. coli* isolates (24.3-fold increase) and between donor *E. coli* isolates and indigenous water bacteria as recipients (8.3-fold increase). Zinc nanoparticles did also increase transformation efficiency. Nevertheless, this effect was nanoparticle-dependent since Zn(NO_3_)_2_ in equivalent concentrations did not increase conjugation frequency or transformation efficiency in the model proposed by the authors (Wang et al., 2018). Similar conjugation experiments showed that copper nanoparticles and CuSO_4_ at 20 and 50 mg/L could reduce the conjugation frequency of catabolic plasmids among *Cupriavidus pinatubonensis*, *Pseudomonas putida*, and *Pseudomonas* sp. isolates by 10% (Parra et al., 2019).

The transconjugants obtained from mating experiments in the presence of ZnSO_4_ and CuSO_4_ did not acquire increased tolerance to any of these metal salts compared to the original recipient strain *E. coli* CV601. These results suggest that our experiments did not involve the co-transfer of ARGs and copper/zinc resistance genes. Additionally, WGS analysis of transconjugants revealed the acquisition of only two genes encoding lithium (*nhaA*) and chromate (*srpC2*) transporters. This indicates a low potential for metals to promote the spread of antibiotic resistance plasmids compatible with *E. coli* in these waterways. Supporting these findings, Pal et al. (2015) reported patterns of genetic co-occurrence of ARGs and biocide/metal resistance genes in bacterial genomes and plasmids from different taxa and environments. The authors identified genes conferring resistance to mercury (*mer* genes), quaternary ammonium compounds (*qacE*Δ*1*) and the class 1 integron integrase intI1 gene, *intl1* as the most common co-occurrence with a wide variety of ARGs in plasmids implying the high probability for co-selection of antibiotic resistance. These results reinforce the findings of several studies that suggest that the presence of *intl1* gene could serve as a marker for anthropogenic pollutants due to its widely spread among pathogenic and commensal bacteria of humans or domestic animals, often located in mobile genetic elements and commonly associated with ARGs, disinfectants and heavy metals (Stalder et al., 2012; Gillings et al., 2015; Agramont et al., 2020; Zheng et al., 2020; Baltazar et al., 2022). The authors reported that cadmium and zinc resistance genes (*cadD*) only co-occurred with aminoglycoside and macrolide resistance genes. According to Pal et al. (2015), silver, copper, and arsenic resistance genes were less likely to co-localize and co-select for other ARGs. The co-occurrence of biocide/metal resistance genes and ARGs was more commonly found in clinical isolates, while this genetic co-localization was less common in plasmids of environmental isolates (<0.7%) (Pal et al., 2015). Finally, the authors showed that plasmids carrying biocide/metal and antibiotic resistance genes tended to be conjugative and carry toxin-antitoxin system genes, promoting the persistence of bacteria and plasmids. In our study, although transconjugants did not acquire zinc and copper resistance genes, many of them acquired multi-drug resistance plasmids carrying ARGs co-localized with genes conferring resistance to biocides such as QACs (*qacA* and *qacE*Δ*1*) and toxin-antitoxin system genes (*dinJ, ldrD*, *mazE*, and *mazF)*.

Most conjugation experiments to test the effect of metals on antibiotic resistance transfer are performed between one specific donor strain (carrying a specific plasmid) and one recipient. To our extent of knowledge, this is the first study of metals at sub-lethal concentrations as stressors in conjugation experiments between complex waterborne bacterial communities as donors and *E. coli* as the recipient. In that way, we were able to evaluate not only the conjugative transfer rate but also the diversity and richness of phenotypic MDRPs and identify the transferred genes and plasmids in the absence and presence of metals by WGS of transconjugants. Significantly higher diversity and richness and unique MDRPs carrying ESBLs such as *bla*_CTX–M–15_, *bla*_SHV–129_, and *bla*_OXA–9_ were obtained only from the agricultural site when metals were added to mating experiments. The agricultural area in the La Paz River is characterized by the production of lettuce, chard, and chamomile, among other crops irrigated with contaminated water from the river. Furthermore, farmers use river sediments as manure for crops. In a previous study, we reported the presence of different categories of diarrheagenic *E. coli* (DEC), *S. enterica*, *K. pneumoniae*, and *Shigella* spp. in soil samples and vegetables from the agricultural area in the La Paz River. *E. coli* isolates carrying ARGs for macrolides and quinolones and ESBLs were also recovered from soil samples (Guzman-Otazo et al., 2019). Soils are considered important reservoirs of antibiotic-resistant bacteria (ARB) and ARGs. In agricultural soils, the continuous discharge of reused wastewater for irrigation, manure, and biosolids promotes the enrichment of antibiotics, ARB, and ARGs, which might be transported vertically deeper into the soil layers or horizontally entering the environment in watersheds and other compartments (Christou et al., 2017). Our results suggest that bacterial donors from the agricultural area in the La Paz River carry higher diversity and richness of transferable MGEs and ARGs than donors from the urban area.

All plasmid-like structures identified in this study belonged to the incompatibility group N (IncN, IncN2, and IncN3). Plasmids belonging to the IncN group are commonly found in and mobilized between, members of the Enterobacterales (Rada et al., 2020; Yamagishi et al., 2020; Sellera et al., 2021). This group of plasmids tends to be self-conjugative, and they are highly associated with antibiotic resistance dispersion since they commonly carry ESBLs, oxacillinases, carbapenemases, quinolone, aminoglycoside, and sulfonamide resistance genes, among others (Carattoli, 2009; Garcia-Fernandez et al., 2011; Dolejska et al., 2013). Several studies of tranconjugation between resident waterborne bacteria using *E. coli* CV601 as recipient identified a dominance of IncN plasmids (Flach et al., 2015; Hutinel et al., 2022) corroborating the results of the present study. However, IncF, IncA/C, IncP, IncN, and other plasmids are commonly found in bacteria from water sources, and the reason for the sole isolation of IncN plasmids in this study needs further analysis.

Transconjugants carrying a high number and varieties of ORGs associated with resistance mechanisms to disinfectants and solvents, metal transporters, biofilm formation, survival in stress conditions, and virulence were mainly obtained from conjugation experiments in the presence of ZnSO_4_ and CuSO_4_. Bacteria and other microorganisms have developed metabolic advantages in the presence of low concentrations of metals. For example, bacteria have mechanisms to transform metals from insoluble to soluble forms and take advantage of metals such as zinc and copper that participate in redox reactions and in the electron transport chain to synthesize more energy molecules and benefit bacterial metabolism (Nguyen et al., 2019). However, heavy metals can also produce oxidative stress, and this has been proposed to be the main mechanism by which metals can promote the spread of antibiotic resistance. The generation of reactive oxygen species (ROS), the activation of SOS response, and the increased permeability of bacterial membranes might promote the horizontal transfer and acquisition of MGEs (Zhang et al., 2018b). Although this study did not show a significant difference in the conjugative transfer rate in the absence and presence of metals, higher diversity, and richness of MDRPs, ARGs, and ORGs were observed in the presence of metals. We might speculate that an energetic and metabolic advantage or the oxidative stress caused by sub-inhibitory concentrations of ZnSO_4_ and CuSO_4_ may favor the transfer/acquisition of a higher diversity of ARGs and ORGs. Assuming that low concentrations of metals promote stress conditions and a significant increase in membrane permeability, this might influence the acquisition of bigger MGEs carrying a higher number and diversity of relevant genes that at the same time confer an advantage to the host in stress conditions. This is the case of unique MDRP P27 and P28 obtained from metal treatment, which contains the highest number of ARGs and ORGs observed in this study. Although the approximate number of base pairs acquired by transconjugants did not significantly differ between sites and treatments, contigs carrying ARGs and ORGs obtained from metal treatment showed an apparent bigger size in base pairs.

In conclusion, this study shows the high potential for a large set of resistance factors to be transferred from polluted bacterial communities in the La Paz River basin to *E. coli*. Metal stressors such as ZnSO_4_ and CuSO_4_ at the sub-lethal concentrations tested in this study did not affect transfer frequencies of antibiotic resistance. However, the presence of metal salts influenced the transfer/acquisition of higher diversity, richness, and unique MDRPs in *E. coli.* This study found the highest diversity of phenotypic MDRPs, ARGs, and ORGs in transconjugants obtained from agricultural water samples in the presence of metal salts during conjugation experiments. Hence, agricultural ecosystems might represent important reservoirs of ARGs and MGEs, posing a risk of transmission of antibiotic resistance to the community by consuming contaminated vegetables. ESBLs and ORGs associated with resistance to disinfectants and antimicrobial peptides, multi-drug transporters, biofilm formation and persisted state in bacteria, survival in stress conditions, virulence determinants and markers for anthropogenic pollution such as the class 1 integron integrase intI1 were also transferred from waterborne bacteria to *E. coli* making evident that contaminated watersheds and ARGs reservoirs in the environment represent a risk for human health.

## Data availability statement

The datasets presented in this study can be found in online repositories. The names of the repository/repositories and accession number(s) can be found in the article/Supplementary material.

## Author contributions

ÅS, DL, VI, and C-FF contributed to the conception and design of the study. JG-O, JA, NM, and VI were involved in the sample collection. JG-O, JJ, and JA performed the experiments. JG-O, EJ, and JA analyzed the data, performed the statistical analysis and visualization. FB and YH performed the bioinformatic analysis. EJ curated the data. JG-O, DL, VI, and ÅS wrote the first draft of the manuscript. AF, DL, EJ, and JA wrote sections of the manuscript. AF, DJ-L, C-FF, and DL edited the manuscript. All authors contributed to manuscript revision, read, and approved the submitted version.

## Footnotes

1
http://www.eucast.org/

2
https://clsi.org/

3
http://bpmsg.com

4
www.CHROMagar.com
