# Supplementary material for: Conjugative transfer of multi-drug resistance IncN plasmids from environmental waterborne bacteria to Escherichia coli
Source: Front Microbiol. 2022 Oct 26;13:997849. doi: 10.3389/fmicb.2022.997849 (PMC9644689; doi:10.3389/fmicb.2022.997849)
Supplement: Supplementary file 1 [file Data_Sheet_1.PDF]

Supplementary Figures

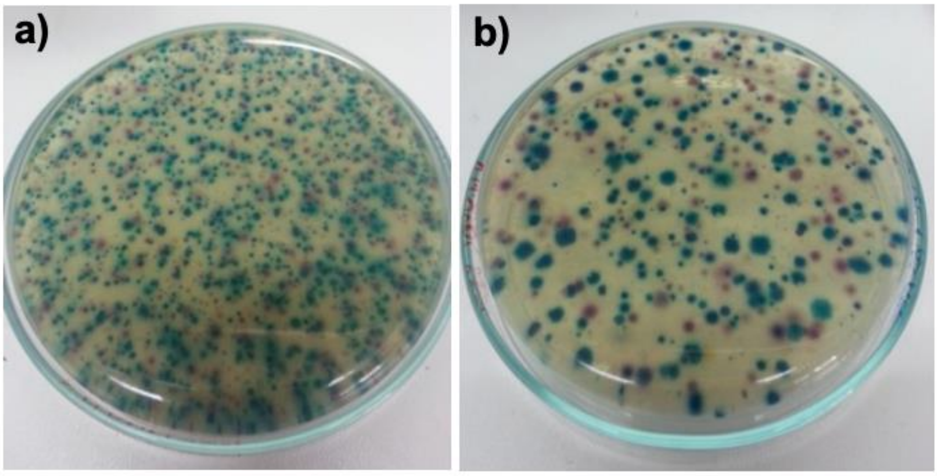

Figure S1. Culturable donor communities in the La Paz River basin (Bolivia). River water samples from the urban (a) and agricultural (b) sampling sites were plated on CHROMagar orientation media at 10^{-2} dilution and incubated for 48 hours at 30°C. Plate reading code according to the manufacturer (www.CHROMagar.com) is *E. coli*: dark pink to reddish; *Enterococcus*: turquoise blue; *Klebsiella*, *Enterobacter*, and *Serratia*: metallic blue; *S. aureus*: golden, opaque, and small and *Pseudomonas aeruginosa*: translucent, cream to blue.

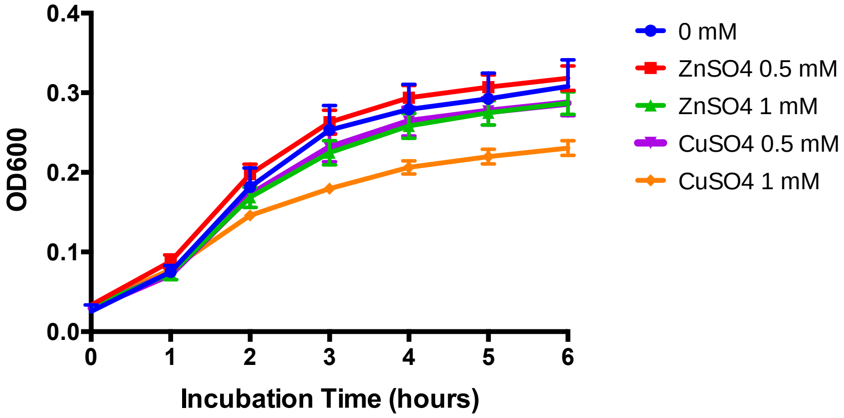

Figure S2. Growth kinetics results for *E. coli* CV601 in the presence of ZnSO4 and CuSO4 at two sub-MIC concentrations. The growth of the recipient strain was measured at 600 nm (OD_{600}) during six hours of incubation at 37°C in the presence of $ZnSO_4x7H_2O$ or $CuSO_4x5H_2O$ at 0.5 and 1 mM. The mean of OD_{600} per hour and per treatment plus the correspondent standard deviation (SD) is shown. Data represent two independent experiments with three replicates each. $CuSO_4$ at 1mM induced a significant decrease in growth (29%) when compared to the control group in the absence of metals (One-way ANOVA $p<0.001$).
