# Supplementary material for: Conjugative transfer of multi-drug resistance IncN plasmids from environmental waterborne bacteria to Escherichia coli
Source: Front Microbiol. 2022 Oct 26;13:997849. doi: 10.3389/fmicb.2022.997849 (PMC9644689; doi:10.3389/fmicb.2022.997849)
Supplement: Supplementary file 2 [file Table_1.DOCX]

# Supplementary Material

1. **Supplementary Tables**

**Table S1.** Comparison between the observed phenotype and genotype in sequenced transconjugants representing the 28 MDRPs obtained in this study.

| **MDRP^a^** | **Treatment^b^** | **Phenotype** | | **Genotype** | | |
| --- | --- | --- | --- | --- | --- | --- |
|  |  | **Resistance** | **Intermediate resistance** | **ARGs^c^**  **Res Finder^e^** | **ARGs^c*^**  **BACTpipe^f^** | **ORGs^d*^**  **BACTpipe^f^** |
| 1 | C  M | SMX-TMP | - | *aadA2, sul1, dfrA1* | *folP1, folP2, dhfrI, ant1* | *emrE1, emrE2, bin3, pld, yafQ1, yafQ2, ldrD* |
| 2* | M | SMX-TMP | NA | *aadA2, sul1, dfrA1* | *folP1, folP2, dhfrI, ant1* | *emrE1, emrE2, bin3, pld, yafQ1, yafQ2, intS1, intS2* |
| 3 | C  M | NA, SMX-TMP | - | *sul1, dfrA25, QnrB2* | *folP1, folP2, dhfrI, qnrB1* | *emrE1, emrE2, bin3, pld, yafQ1, yafQ2, pspF1, pspF2, sapA1, sapA2* |
| 4 (U) | M | SMX-TMP | STR | *aadA2, sul1, dfrA1* | *folP1, folP2, dhfrI, ant1* | *emrE1, emrE2, bin3, pld, yafQ1, yafQ2* |
| 5 | M | AMP, SMX-TMP | NA | *sul1, dfrA16, bla*_TEM_*_-_*_1B_*, QnrS1* | *folP1, folP2, dhfrI, qnrB1, bla_TEM_* | *emrE1, emrE2, bin3, pld, tnpR* |
| 6 | C  M | AMP, NA, SMX-TMP | - | *sul1, dfrA16, bla*_TEM_*_-_*_1B_*, QnrS1* | *folP1, folP2, dhfrI, qnrB1, bla*_TEM_ | *emrE1, emrE2, bin3, pld, tnpR* |
| 7 (U) | M | AMP, SMX-TMP | STR | *aadA2, sul1, dfrA1, bla*_TEM_*_-_*_1A_ | *folP1, folP2, dhfrI, ant1, bla*_TEM_ | *emrE1, emrE2, bin3, pld, yafQ1, yafQ2, tnpR* |
| 8 | C  M | AMP, STR, SMX-TMP | - | *strA, strB-like, sul2, dfrA14-like bla*_TEM_*_-_*_1B_*_,_ tet(D)-like* | *folP1, folP2, dhfrI, bla*_TEM_*, neo, tetA, tetR* | *pld, tnpR* |
| 9 | M | NA, SMX-TMP | STR | *aadA2, sul1, dfrA1, QnrB2* | *folP1, folP2, folP3, dhfrI, ant1, qnrB1* | *emrE1, emrE2, bin3, pld, yafQ1, yafQ2, pspF1, pspF2, sapA1, sapA2* |
| 10 | C  M | TE, NA, SMX-TMP | - | *sul1, dfrA25, QnrB2, tet(A)-like* | *folP1, folP2, folP3, dhfrI, qnrB1, tetA, tetR* | *emrE1, emrE2, pld, pspF1, pspF2, sapA1, sapA2, yedA1, yedA2* |
| 11 (U) | M | AMP, CTX, SMX-TMP | - | *sul1, dfrB4, bla*_CTX_*_-_*_M-15_*, bla*_TEM-1B_*, qepA, mph(A)* | *folP1, folP2, folA, bla*_TEM_*,*  *bla*_CTX-M-1_ | *emrE1, emrE2, pld, tnpR, groL1, groL2, mazE1, mazE2, mazF1, mazF2, qacA, srpC1, srpC2, ynaI1, ynaI2, Rv1258c* |
| 12 (U) | M | CIP, NA, SMX-TMP | - | *aadA16-like, sul1, dfrA27, QnrB6, ARR-3, aac(6')-Ib-cr* | *folP1, folP2, folP3, dhfrI, ant1, qnrB1, aacA4* | *emrE1, emrE2, emrE3, pld, pspF1, pspF2* |
| 13 | M | AMP, TE, SMX-TMP | STR | *strA, sul2, dfrA14-like, bla*_TEM-1B_*, tet(D)* | *folP1, folP2, dhfrI, bla*_TEM_*, neo, tetA, tetR* | *bin3, pld, tnpR, nhaA1, nhaA2, pinR1, pinR2, ldrD* |
| 14 | C  M | AMP, TE, STR, SMX-TMP | - | *strA, strB, sul2, dfrA14-like, bla*_TEM-1B_*, tet(D)* | *folP1, folP2, dhfrI, bla*_TEM_*, neo, tetA, tetR* | *bin3, pld, tnpR, nhaA1, nhaA2, pinR1, pinR2* |
| 15 | M | AMP, STR, SMX-TMP | NA | *strA, strB, sul2, dfrA14-like, bla*_TEM-1B_*, QnrS1* | *folP1, folP2, dhfrI, bla*_TEM_*, neo, qnrB1, ftsI1, ftsI2* | *pld, tnpR* |
| 16 | C  M | AMP, NA, STR, SMX-TMP | - | *strA, strB, sul2, dfrA14-like, QnrS1* | *folP1, folP2, dhfrI, bla*_TEM_*, neo, qnrB1, ftsI1, ftsI2* | *pld* |
| 17 | C  M | AMP, NA, SMX-TMP | CIP | *sul1, dfrA16, bla*_TEM-1B_*, QnrS1* | *folP1, folP2, dhfrI, qnrB1, bla*_TEM_ | *emrE1, emrE2, bin3, pld, tnpR* |
| 18 (U)* | M | AMP, CTX, SMX-TMP | NA | *aadA2, sul1, dfrA1, bla*_SHV-129_ | *folP1, folP2, dhfrI, ant1, bla*_SHV-2_ | *emrE1, emrE2, bin3, pld, yafQ1, yafQ2* |
| 19 (U) | M | TE, NA, SMX-TMP | CIP | *aadA16-like, sul1, dfrA27, QnrB6, tet(A)-like, ARR-3, aac(6')-Ib-cr* | *folP1, folP2, dhfrI, ant1, qnrB1, tetA, tetR, aacA4* | *emrE1, emrE2, pld, pspF1, pspF2, yedA1, yedA2* |
| 20 (U) | M | AMP, NA, SMX-TMP | CTX | *sul1, dfrA25, bla*_SHV-129_*, QnrB2* | *folP1, folP2, folP3, dhfrI, qnrB1, bla*_SHV-2_ | *emrE1, emrE2, bin3, pld, yafQ1, yafQ2, pspF1, pspF2, sapA1, sapA2* |
| 21 | M | AMP, NA, STR, SMX-TMP | CIP | *strA, strB, sul1, sul2, dfrA16, bla*_TEM-1B_*, QnrS1* | *folP1, folP2, folP3, dhfrI, bla*_TEM_*, neo, qnrB1, ftsI1, ftsI2* | *emrE1, emrE2, bin3, pld1, pld2, tnpR* |
| 22 | C  M | AMP, TE, STR, SMX-TMP | NA | *strA, strB, sul2, dfrA14-like, bla*_TEM-1B_*_,_ tet(D)* | *folP1, folP2, dhfrI, bla*_TEM_*, neo, tetA, tetR* | *bin3, pld, tnpR, nhaA1, nhaA2, pinR1, pinR2* |
| 23 (U) | M | AMP, STR, SMX-TMP | CIP, NA | *strA, strB, sul2, dfrA14-like, QnrS1* | *folP1, folP2, dhfrI, bla*_TEM_*, neo, qnrB1, ftsI1, ftsI2* | *pld* |
| 24 (U) | M | AMP, CHL, NA, SMX-TMP | CIP | *sul1, dfrA16, bla*_TEM-1B_*, QnrS1* | *folP1, folP2, dhfrI, qnrB1, bla*_TEM_ | *emrE1, emrE2, bin3, pld, tnpR* |
| 25 (U) | M | AMP, CTX, NA, SMX-TMP | STR | *aadA2, sul1, dfrA1, bla*_SHV-129_ | *folP1, folP2, dhfrI, ant1, bla*_SHV-2_ | *emrE1, emrE2, bin3, pld, yafQ1, yafQ2* |
| 26 (U) | M | AMP, TE, STR, SMX-TMP | TZP | *strA, sul2, dfrA14-like, bla*_TEM-1B_*, tet(D)* | *folP1, folP2, dhfrI, bla*_TEM_*, neo, tetA, tetR* | *bin3, pld, tnpR, nhaA1, nhaA2, pinR1, pinR2* |
| 27 (U) | M | AMP, TE, NA, STR, SMX-TMP | TZP | *strA, strB, sul1, sul2, dfrA14-like, dfrA16, bla*_TEM-1B_*, QnrS1, tet(D)* | *folP1, folP2, folP3, dhfrI1, dhfrI2, qnrB1, bla*_TEM_*, neo, tetA, tetR* | *emrE1, emrE2, bin3.1, bin3.2, pld1, pld2, tnpR, nhaA1, nhaA2, pinR1, pinR2, yafQ1, yafQ2, dinJ1, dinJ2, hha1, hha2, ptlE, topB1, topB2, traG* |
| 28 (U) | M | AMP, TE, CIP, NA, STR, SMX-TMP | - | *aac(6')-Ib-like, aadA1, aadA2-like, strA, sul1, sul2, dfrA1, dfrA14-like, bla*_OXA-9_*, bla*_TEM-1B_*_,_ QnrB2, tet(D), aac(6')Ib-cr-like* | *folP1, folP2, folP3, dhfrI1, dhfrI2, ant1.1, ant1.2, qnrB1, bla*_TEM_*, bla*_OXA_*, neo, tetA, tetR, aacA4* | *bin3.1, bin3.2, pld1, pld2, tnpR, nhaA1, nhaA2, pinR1, pinR2, yafQ1, yafQ2, pspF1, pspF2, sapA1, sapA2* |

^a^ Multi-drug resistance profile (MDRP); U= unique profile in the study.

^b^ Treatment from where the profile was obtained C= control treatment, LB media without metals (0 mM); M= metal treatment, LB media supplemented with ZnSO4 or CuSO4 at 0.5 or 1 mM.

^c^ Antibiotic resistance genes (ARGs)

^d^ Other relevant genes (ORGs)

*Consecutive numbers (1, 2, 3) are used when two or more copies of one specific gene are present.

^e^ Identification and annotation of genes using Resistance Finder database

^f^ Identification and annotation of genes using BACTpipe database

Antibiotics: ampicillin, AMP; tetracycline, TET; ciprofloxacin, CIP; chloramphenicol, CHL; cefotaxime, CTX; meropenem, MEM; doripenem, DOR; ertapenem, ETP; imipenem, IMI; nalidixic acid, NA; gentamicin, CN; streptomycin, STR; piperacillin-tazobactam, PTZ and sulfamethoxazol/trimethoprim SMX/TMP.

**Table S2.** Key features of the whole genome sequenced transconjugants obtained in this study.

| **Isolate** | **Treatment** | **MDRP^a^** | **Genome size (bp)^b^** | **DNA transferred (bp)^b^** | **RSGs^c^** | | **SRGs^d^** | **BRGs BacMete** | **Class 1 integron** | **Plasmidf Inc group** | **pMLST^g^** | |
| --- | --- | --- | --- | --- | --- | --- | --- | --- | --- | --- | --- | --- |
| *E. coli* CV601 |  |  | 4583870 |  | |  |  |  |  |  | |  |
| **Urban** | | | | | | | | | | | | |
| USA-1-3 | LB | P14 | 4668342 | 84472 | *ECORII, dcm* | | *sul2* | NF | *intl1* | IncN | ST-6 | |
| USA-1-8 |  | P1 | 4624130 | 40260 | *klcA2* | | *sul1* | *qacE*, *qacED1* | *intl1* | IncN3 | Unknown | |
| USA-1-13 |  | P16 | 4642637 | 58767 | *klcA2, ECORII, dcm* | | *sul2* | NF | *intl1* | IncN | ST-6 | |
| USA-1-20 |  | P6 | 4638136 | 54266 | *hsdR, hsdM* | | *sul1* | *qacE*, *qacED1* | *intl1* | IncN2 | Unknown | |
| USA-2-1 |  | P3 | 4631144 | 47274 | *klcA2* | | *sul1* | *qacE*, *qacED1* | *intl1* | IncN3 | Unknown | |
| USA-2-4 |  | P17 | 4637663 | 53793 | *hsdR, hsdM* | | *sul1* | *qacE*, *qacED1* | *intl1* | IncN2 | Unknown | |
| USB-1-1 | LB+ZnSO_4_ 0.5 mM | P21 | 4685988 | 102118 | *NF* | | *sul1, sul2* | *qacE*, *qacED1* | *intl1* | IncN2 | ST-6 | |
|  |  |  |  |  |  |  |  |  | *intl1* | IncN |  |  |
| USB-1-18 |  | P22 | 4659754 | 75884 | *ECORII, dcm* | | *sul2* | NF | *intl1* | IncN | ST-6 | |
| USB-2-3 |  | P13 | 4661026 | 77156 | *ECORII, dcm* | | *sul2* | NF | *intl1* | IncN | ST-6 | |
| USC-1-11 | LB+ZnSO_4_ 1 mM | P2 | 4624106 | 40236 | *klcA2* | | *sul1* | *qacE*, *qacED1* | *intl1* | IncN3 | Unknown | |
| USC-1-14 |  | P16 | 4640647 | 56777 | *klcA2, ECORII, dcm* | | *sul2* | NF | *intl1* | IncN | ST-6 | |
| USC-2-12 |  | P23 | 4642081 | 58211 | *klcA2, ECORII, dcm* | | *sul2* | NF | *intl1* | IncN | ST-6 | |
| USD-1-4 | LB+CuSO_4_ 0.5 mM | P15 | 4634607 | 50737 | *klcA2, ECORII, dcm* | | *sul2* | NF | *intl1* | IncN | ST-6 | |
| USD-1-11 |  | P14 | 4661074 | 77204 | *ECORII, dcm* | | *sul2* | NF | *intl1* | IncN | ST-6 | |
| USD-1-19 |  | P17 | 4637950 | 54080 | *hsdR, hsdM* | | *sul1* | *qacE*, *qacED1* | *intl1* | IncN2 | Unknown | |
| USD-2-19 |  | P5 | 4638190 | 54320 | *hsdR, hsdM* | | *sul1* | *qacE*, *qacED1* | *intl1* | IncN2 | Unknown | |
| USE-2-18 | LB+CuSO_4_ 1 mM | P24 | 4639067 | 55197 | *hsdR, hsdM* | | *sul1* | *qacE*, *qacED1* | *intl1* | IncN2 | Unknown | |
| USE-2-20 |  | P6 | 4636546 | 52676 | *hsdR, hsdM* | | *sul1* | *qacE*, *qacED1* | *intl1* | IncN2 | Unknown | |
| **Agriculture** | | | | | | | | | | | | |
| ASA-1-3 | LB | P6 | 4637682 | 53812 | *hsdR, hsdM* | | *sul1* | *qacE*, *qacED1* | *intl1* | IncN2 | Unknown | |
| ASA-1-8 |  | P22 | 4641970 | 58100 | *ECORII, dcm* | | *sul2* | NF | *intl1* | IncN | ST-6 | |
| ASA-1-12 |  | P16 | 4642488 | 58618 | *klcA2, ECORII, dcm* | | *sul2* | NF | *intl1* | IncN | ST-6 | |
| ASA-2-3 |  | P8 | 4636304 | 52434 | *ECORII, dcm* | | *sul2* | NF | *intl1* | IncN | ST-6 | |
| ASB-1-11 | LB+ZnSO_4_ 0.5 mM | P18 | 4634229 | 50359 | *klcA2* | | *sul1* | *qacE*, *qacED1* | *intl1* | IncN3 | Unknown | |
| ASB-1-18 |  | P28 | 4710972 | 127102 | *NF* | | *sul1, sul2* | *qacE*, *qacED1* | *intl1* | IncN3 | ST-6 | |
|  |  |  |  |  |  |  |  |  |  | IncN |  |  |
| ASB-2-1 |  | P13 | 4662799 | 78929 | *ECORII, dcm* | | *sul2* | NF | *intl1* | IncN | ST-6 | |
| ASB-2-13 |  | P14 | 4663737 | 79867 | *ECORII, dcm* | | *sul2* | NF | *intl1* | IncN | ST-6 | |
| ASC-1-5 | LB+ZnSO_4_ 1 mM | P6 | 4637682 | 53812 | *hsdR, hsdM* | | *sul1* | *qacE*, *qacED1* | *intl1* | IncN2 | Unknown | |
| ASC-1-9 |  | P7 | 4629591 | 45721 | *klcA2* | | *sul1* | *qacE*, *qacED1* | *intl1* | IncN3 | Unknown | |
| ASC-1-19 |  | P21 | 4642022 | 58152 | *klcA2, ECORII, dcm* | | *sul2* | NF | *intl1* | IncN | ST-6 | |
| ASC-2-2 |  | P15 | 4642166 | 58296 | *klcA2, ECORII, dcm* | | *sul2* | NF | *intl1* | IncN | ST-6 | |
| ASC-2-7 |  | P5 | 4637611 | 53741 | *hsdR, hsdM* | | *sul1* | *qacE*, *qacED1* | *intl1* | IncN2 | Unknown | |
| ASC-2-18 |  | P1 | 4624997 | 41127 | *klcA2* | | *sul1* | *qacE*, *qacED1* | *intl1* | IncN3 | Unknown | |
| ASC-3-4 |  | P25 | 4634186 | 50316 | *klcA2* | | *sul1* | *qacE*, *qacED1* | *intl1* | IncN3 | Unknown | |
| ASC-3-9 |  | P10 | 4633787 | 49917 | *ECORII, dcm* | | *sul1* | *qacE*, *qacED1* | *intl1* | IncN | ST-5 | |
| ASD-1-5 | LB+CuSO_4_ 0.5 mM | P9 | 4630508 | 46638 | *klcA2* | | *sul1* | *qacE*, *qacED1* | *intl1* | IncN3 | Unknown | |
| ASD-1-10 |  | P2 | 4645671 | 61801 | *klcA2* | | *sul1* | *qacE*, *qacED1* | *intl1* | IncN3 | Unknown | |
| ASD-1-13 |  | P19 | 4636751 | 52881 | *klcA2, ECORII, dcm* | | *sul1* | *qacE*, *qacED1* | *intl1* | IncN | ST-5 | |
| ASD-2-4 |  | P8 | 4641272 | 57402 | *ECORII, dcm* | | *sul2* | NF | *intl1* | IncN | ST-6 | |
| ASD-2-15 |  | P16 | 4641452 | 57582 | *klcA2, ECORII, dcm* | | *sul2* | NF | *intl1* | IncN | ST-6 | |
| ASD-3-1 |  | P11 | 4648881 | 65011 | *klcA2* | | *sul1* | *qacE*, *qacED1* | *intl1* | IncN3 | Unknown | |
| ASD-3-2 |  | P12 | 4635349 | 51479 | *ECORII, dcm* | | *sul1* | *qacE*, *qacED1* | *intl1* | IncN | ST-5 | |
| ASE-1-3 | LB+CuSO_4_ 1 mM | P17 | 4638742 | 54872 | *hsdR, hsdM* | | *sul1* | *qacE*, *qacED1* | *intl1* | IncN2 | Unknown | |
| ASE-1-5 |  | P22 | 4642372 | 58502 | *ECORII, dcm* | | *sul2* | NF | *intl1* | IncN | ST-6 | |
| ASE-1-18 |  | P20 | 4639525 | 55655 | *klcA2* | | *sul1* | *qacE*, *qacED1* | *intl1* | IncN3 | Unknown | |
| ASE-3-3 |  | P27 | 4747945 | 164075 | *klcA2, hsdR, hsdM* | | *sul1, sul2* | *qacE*, *qacED1* | *intl1* | IncN2 | ST-6 | |
|  |  |  |  |  |  |  |  |  |  | IncN |  |  |
|  |  |  |  |  |  |  |  |  |  | pSL483 |  |  |
| ASE-3-6 |  | P26 | 4660905 | 77035 | *ECORII, dcm* | | *sul2* | NF | *intl1* | IncN | ST-6 | |
| ASE-3-8 |  | P4 | 4622680 | 38810 | *klcA2* | | *sul1* | *qacE*, *qacED1* | *intl1* | IncN3 | Unknown | |

^a^ Multi-drug resistance profile (MDRP);

^b^ Approximate genome size and DNA transferred in base pairs;

^c^ Restriction system genes (RSGs); NF= not found ;

^d^ Sulfonamide resistance gene (SRGs);

^e^ Biocide resistance genes (BRGs) acquired by transconjugants were identified using BacMet database. NF= not found means that sequenced transconjugant carries the same set of biocide and metal-like resistance genes as the recipient.;

^f^ Plasmid Incompatibility group (Inc group);

^g^ Plasmid multi-locus sequence type (pMLST)

**Table S3.** Biocide- and metal-like resistance genes found in the recipient strain of *Escherichia coli* CV601 genome by sequence

analysis using BacMet database.

| **N°** | **BacMet ID** | **Gene name** | **Organism** | **Location** | **Compound** |
| --- | --- | --- | --- | --- | --- |
| 1 | BAC0006 | *acrB* | *Escherichia coli* (strain K12) | Chromosome | Acriflavine [class: Acridine], Phenol [class: Phenolic compounds], Triclosan [class: Phenolic compounds], p-xylene [class: Aromatic hydrocarbons], Cyclohexane [class: Cycloalkane], Pentane [class: Alkane] |
| 2 | BAC0008 | *acrD/yffA* | *Escherichia coli* (strain K12) | Chromosome | Sodium Dodecyl Sulfate (SDS) [class: Organo-sulfate], Sodium Deoxycholate (SDC) [class: Acid] |
| 3 | BAC0010 | *acrF/envD* | *Escherichia coli* (strain K12) | Chromosome | Acriflavine [class: Acridine], Sodium Dodecyl Sulfate (SDS) [class: Organo-sulfate], Sodium Deoxycholate (SDC) [class: Acid], Tetraphenylphosphonium (TPP) [class: Quaternary Ammonium Compounds (QACs)], Benzylkonium Chloride (BAC) [class: Quaternary Ammonium Compounds (QACs)], Methyl Viologen [class: Paraquat], Ethidium Bromide [class: Phenanthridine] |
| 4 | BAC0031 | *arsB* | *Escherichia coli* | Plasmid R773 (E. coli), Plasmid IncN R46 (E. coli), Plasmid R46 (Salmonella typhimurium) | Arsenic (As), Antimony (Sb) |
| 5 | BAC0039 | *baeR* | *Escherichia coli* (strain K12) | Chromosome | Zinc (Zn), Tungsten (W), Sodium Deoxycholate (SDC) [class: Acid] |
| 6 | BAC0040 | *baeS* | *Escherichia coli* (strain K12) | Chromosome | Zinc (Zn), Tungsten (W), Sodium Deoxycholate (SDC) [class: Acid] |
| 7 | BAC0041 | *bcr* | *Escherichia coli* (strain K12) | Chromosome | Acriflavine [class: Acridine] |
| 8 | BAC0049 | *bhsA/ycfR/comC* | *Escherichia coli* (strain K12) | Chromosome | Copper (Cu) |
| 9 | BAC0076 | *comR/ycfQ* | *Escherichia coli* (strain K12) | Chromosome | Copper (Cu) |
| 10 | BAC0086 | *corA* | *Escherichia coli* (strain K12) | Chromosome | Magnesium (Mg), Cobalt (Co), Nickel (Ni), Manganese (Mn) |
| 11 | BAC0087 | *mgtA* | *Escherichia coli* (strain K12) | Chromosome | Cobalt (Co), Magnesium (Mg) |
| 12 | BAC0088 | *corC* | *Salmonella typhimurium*  (strain LT2 / SGSC1412 / ATCC 700720) | Chromosome | Cobalt (Co), Magnesium (Mg) |
| 13 | BAC0103 | *cueO* | *Escherichia coli* (strain K12) | Chromosome | Copper (Cu) |
| 14 | BAC0107 | *cusA/ybdE* | *Escherichia coli* (strain K12) | Plasmid megaplasmid CH34 (Ralstonia metallidurans (strain CH34 / ATCC 43123 / DSM 2839)), | Copper (Cu), Silver (Ag) |
| 15 | BAC0108 | *cusB* | *Ralstonia metallidurans*  (strain CH34 / ATCC 43123 / DSM 2839) | Plasmid megaplasmid CH34 (Ralstonia metallidurans (strain CH34 / ATCC 43123 / DSM 2839)), | Copper (Cu), Silver (Ag) |
| 16 | BAC0109 | *cusC/ylcB* | *Escherichia coli* (strain K12) | Plasmid megaplasmid CH34 (Ralstonia metallidurans (strain CH34 / ATCC 43123 / DSM 2839)) | Copper (Cu), Silver (Ag) |
| 17 | BAC0111 | *cusR/ylcA* | *Escherichia coli* (strain K12) | Plasmid pECL_A (Enterobacter cloacae subsp. cloacae (strain ATCC 13047 / DSM 30054 / NBRC 13535 / NCDC 279-56)) | Copper (Cu), Silver (Ag) |
| 18 | BAC0112 | *cusS* | *Escherichia coli* (strain K12) | Chromosome | Copper (Cu), Silver (Ag) |
| 19 | BAC0113 | *cutA* | *Escherichia coli* (strain K12) | Chromosome | Copper (Cu) |
| 20 | BAC0114 | *cutC* | *Escherichia coli* (strain K12) | Chromosome | Copper (Cu) |
| 21 | BAC0115 | *cutE/lnt* | *Escherichia coli* (strain K12) | Chromosome | Copper (Cu) |
| 22 | BAC0116 | *cutF/nlpE* | *Escherichia coli* (strain K12) | Chromosome | Copper (Cu) |
| 23 | BAC0136 | *dsbA* | *Escherichia coli* (strain K12) | Chromosome | Cadmium (Cd), Zinc (Zn), Mercury (Hg) |
| 24 | BAC0137 | *dsbB* | *Escherichia coli* (strain K12) | Chromosome | Cadmium (Cd), Mercury (Hg) |
| 25 | BAC0138 | *dsbC* | *Escherichia coli* (strain K12) | Chromosome | Copper (Cu) |
| 26 | BAC0147 | *emrA* | *Escherichia coli* (strain K12) | Chromosome | Phenylmercury Acetate [class: Organo-mercury], 2-Chlorophenylhydrazine [class: Hydrazine], Carbonylcyanide m-chlorophenyl hydrazone (CCCP) [class: Hydrazone], Tetrachlorosalicylanilide (TCS) [class: Salicylanilide] |
| 27 | BAC0148 | *emrB* | *Escherichia coli* (strain K12) | Chromosome | Phenylmercury Acetate [class: Organo-mercury], 2-Chlorophenylhydrazine [class: Hydrazine], Carbonylcyanide m-chlorophenyl hydrazone (CCCP) [class: Hydrazone] |
| 28 | BAC0149 | *emrD* | *Escherichia coli* (strain K12) | Chromosome | Benzylkonium Chloride (BAC) [class: Quaternary Ammonium Compounds (QACs)], Sodium Dodecyl Sulfate (SDS) [class: Organo-sulfate] |
| 29 | BAC0150 | *emrE/mvrC* | *Escherichia coli* (Strain K12) | Chromosome | Benzylkonium Chloride (BAC) [class: Quaternary Ammonium Compounds (QACs)], Methyl Viologen [class: Paraquat], Ethidium Bromide [class: Phenanthridine], Tetraphenylphosphonium (TPP) [class: Quaternary Ammonium Compounds (QACs)] |
| 30 | BAC0151 | *emrK* | *Escherichia coli* (strain K12) | Chromosome | Benzylkonium Chloride (BAC) [class: Quaternary Ammonium Compounds (QACs)], Crystal Violet [class: Triarylmethane], Tetraphenylphosphonium (TPP) [class: Quaternary Ammonium Compounds (QACs)], Sodium Deoxycholate (SDC) [class: Acid], Sodium Dodecyl Sulfate (SDS) [class: Organo-sulfate] |
| 31 | BAC0153 | *emrY* | *Escherichia coli* (strain K12) | Chromosome | Sodium Dodecyl Sulfate (SDS) [class: Organo-sulfate], Ethidium Bromide [class: Phenanthridine] |
| 32 | BAC0154 | *evgA* | Only gram (-), E*scherichia coli* (Strain K12) | Chromosome | Sodium Deoxycholate (SDC) [class: Acid] |
| 33 | BAC0155 | *evgS* | *Escherichia coli* (strain K12) | Chromosome | Sodium Deoxycholate (SDC) [class: Acid] |
| 34 | BAC0156 | *fabI* | *Escherichia coli* (strain K12) | Chromosome | Triclosan [class: Phenolic compounds] |
| 35 | BAC0163 | *fecD* | *Escherichia coli* (strain K12) | Chromosome | Nickel (Ni), Cobalt (Co) |
| 36 | BAC0164 | *fecE* | *Escherichia coli* (strain K12) | Chromosome | Nickel (Ni), Cobalt (Co) |
| 37 | BAC0167 | *fieF/yiip* | *Escherichia coli* (strain K12) | Chromosome | Iron (Fe), Zinc (Zn), Cobalt (Co), Cadmium (Cd), Nickel (Ni) |
| 38 | BAC0172 | *gadA* | *Escherichia coli* (strain K12) | Chromosome | Hydrochloric acid (HCl) [class: Acid] |
| 39 | BAC0173 | *gadB* | *Escherichia coli* (strain K12) | Chromosome | Hydrochloric acid (HCl) [class: Acid] |
| 40 | BAC0174 | *gadC/xasA* | *Escherichia coli* (strain K12) | Chromosome | Hydrochloric acid (HCl) [class: Acid] |
| 41 | BAC0181 | *glpF* | *Escherichia coli* (strain K12) | Chromosome | Antimony (Sb), Arsenic (As), Glycerol [class: Alcohol] |
| 42 | BAC0194 | *ibpA* | *Escherichia coli* (strain K12) | Chromosome | Hydrogen Peroxide (H2O2) [class: Peroxides] |
| 43 | BAC0195 | *ibpB* | *Escherichia coli* (strain K12) | Chromosome | Hydrogen Peroxide (H2O2) [class: Peroxides] |
| 44 | BAC0196 | *iclR* | *Escherichia coli* (strain K12) | Chromosome | Sodium acetate [class: Acetate] |
| 45 | BAC0208 | *mdfA/cmr* | Only gram (-), *E. coli* (Strain K12) | Chromosome | Benzylkonium Chloride (BAC) [class: Quaternary Ammonium Compounds (QACs)], Ethidium Bromide [class: Phenanthridine], Acriflavine [class: Acridine], Tetraphenylphosphonium (TPP) [class: Quaternary Ammonium Compounds (QACs)], Sodium Hydroxide (NaOH) [class: Base], Rhodamine 6G [class: Xanthene] |
| 46 | BAC0210 | *mdtA/yegM* | *Escherichia coli* (strain K12) | Chromosome | Sodium Deoxycholate (SDC) [class: Acid] |
| 47 | BAC0211 | *mdtB/yegN* | *Escherichia coli* (strain K12) | Chromosome | Sodium Deoxycholate (SDC) [class: Acid], Hydrochloric acid (HCl) [class: Acid] |
| 48 | BAC0212 | *mdtC/yegO* | *Escherichia coli* (strain K12) | Chromosome | Sodium Deoxycholate (SDC) [class: Acid] |
| 49 | BAC0214 | *mdtF/yhiV* | *Escherichia coli* (strain K12) | Chromosome | Crystal Violet [class: Triarylmethane], Sodium Dodecyl Sulfate (SDS) [class: Organo-sulfate], Tetraphenylphosphonium (TPP) [class: Quaternary Ammonium Compounds (QACs)], Sodium Deoxycholate (SDC) [class: Acid], Rhodamine 6G [class: Xanthene], Benzylkonium Chloride (BAC) [class: Quaternary Ammonium Compounds (QACs)] |
| 50 | BAC0215 | *mdtG/yceE* | *Escherichia coli* (strain K12) | Chromosome | Sodium Deoxycholate (SDC) [class: Acid] |
| 51 | BAC0216 | *mdtI/ydgE* | *Escherichia coli* (strain K12) | Chromosome | Sodium Deoxycholate (SDC) [class: Acid], Sodium Dodecyl Sulfate (SDS) [class: Organo-sulfate], Spermidine [class: Polyamines] |
| 52 | BAC0217 | *mdtJ/ebrB/ydgF* | *Shigella sonnei* (strain Ss046) | Chromosome | Spermidine [class: Polyamines] |
| 53 | BAC0218 | *mdtK/ydhE* | *Escherichia coli* (strain K12) | Chromosome | Tetraphenylphosphonium (TPP) [class: Quaternary Ammonium Compounds (QACs)], Sodium Deoxycholate (SDC) [class: Acid], Ethidium Bromide [class: Phenanthridine], Benzylkonium Chloride (BAC) [class: Quaternary Ammonium Compounds (QACs)], Acriflavine [class: Acridine] |
| 54 | BAC0219 | *mdtM/yjiO* | *Escherichia coli* (strain K12) | Chromosome | Tetraphenylphosphonium (TPP) [class: Quaternary Ammonium Compounds (QACs)], Ethidium Bromide [class: Phenanthridine], Acriflavine [class: Acridine], Tetraphenylarsonium (TPA) [class: Quaternary Ammonium Compounds (QACs)] |
| 55 | BAC0220 | *mdtN/yjcR* | *Escherichia coli* (strain K12) | Chromosome | Acriflavine [class: Acridine], Tetraphenylarsonium (TPA) [class: Quaternary Ammonium Compounds (QACs)] |
| 56 | BAC0251 | *mntH/yfeP* | *Escherichia coli* (strain K12) | Chromosome | Manganese (Mn), Iron (Fe), Cadmium (Cd), Cobalt (Co), Zinc (Zn) |
| 57 | BAC0252 | *mntP/yebN* | *Escherichia coli* K-12 | Chromosome | Manganese (Mn), Magnesium (Mg), Methyl Viologen [class: Paraquat], Hydrogen Peroxide (H2O2) [class: Peroxides] |
| 58 | BAC0253 | *mntR* | *Escherichia coli* | Chromosome | Manganese (Mn), Magnesium (Mg) |
| 59 | BAC0270 | *nikA* | *Escherichia coli* (strain K12) | Chromosome | Nickel (Ni) |
| 60 | BAC0271 | *nikB* | *Escherichia coli* (strain K12) | Chromosome | Nickel (Ni) |
| 61 | BAC0272 | *nikC* | *Escherichia coli* (strain K12) | Chromosome | Nickel (Ni) |
| 62 | BAC0273 | *nikD* | *Escherichia coli* (strain K12) | Chromosome | Nickel (Ni) |
| 63 | BAC0274 | *nikE* | *Escherichia coli* (strain K12) | Chromosome | Nickel (Ni) |
| 64 | BAC0296 | *ostA/lptD* | *Escherichia coli* (strain K12) | Chromosome | n-hexane [class: Alkane] |
| 65 | BAC0312 | *pitA* | *Escherichia coli* (strain K12) | Chromosome | Zinc (Zn), Tellurium (Te) |
| 66 | BAC0315 | *pstA* | *Escherichia coli* (strain K12) | Chromosome | Arsenic (As) |
| 67 | BAC0316 | *pstB* | *Escherichia coli* (strain K12) | Chromosome | Arsenic (As) |
| 68 | BAC0317 | *pstC* | *Escherichia coli* (strain K12) | Chromosome | Arsenic (As) |
| 69 | BAC0318 | *pstS* | *Escherichia coli* (strain K12) | Chromosome | Arsenic (As) |
| 70 | BAC0330 | *rcnA/yohM* | *Escherichia coli* (strain K12) | Chromosome | Cobalt (Co), Nickel (Ni), Iron (Fe) |
| 71 | BAC0331 | *rcnB/yohN* | *Escherichia coli* (strain K12) | Chromosome | Nickel (Ni), Cobalt (Co) |
| 72 | BAC0332 | *rcnR/yohL* | *Escherichia coli* (strain K12) | Chromosome | Cobalt (Co), Nickel (Ni), Iron (Fe) |
| 73 | BAC0334 | *robA* | *Escherichia coli* O157:H7 | Chromosome | Silver (Ag), Mercury (Hg), Cadmium (Cd), Cyclohexane [class: Cycloalkane], Pentane [class: Alkane], n-hexane [class: Alkane], Diphenyl Ether [class: Phenyl] |
| 74 | BAC0335 | *rpoS* | *Escherichia coli*  *Shigella flexneri* | Chromosome | Hydrochloric acid (HCl) [class: Acid], Sodium hydroxide (NaOH) [class: Base] |
| 75 | BAC0368 | *sodA* | *Escherichia coli* K12 | Chromosome | Selenium (Se), Hydrogen Peroxide (H2O2) [class: Peroxides] |
| 76 | BAC0370 | *soxR* | *Escherichia coli* (strain K12) | Chromosome | Methyl Viologen [class: Paraquat] |
| 77 | BAC0371 | *soxS* | *Escherichia coli* (strain K12) | Chromosome | Zinc (Zn), Cyclohexane [class: Cycloalkane] |
| 78 | BAC0378 | *sugE* | only gram (-), *Escherichia coli* | Plasmid IncN p541 (Escherichia coli), Plasmid pIW759 (Salmonella typhimurium) | Cetrimide (CTM) [class: Quaternary Ammonium Compounds (QACs)], Cetylpyridinium Chloride (CPC) [class: Quaternary Ammonium Compounds (QACs)], Tetraphenylphosphonium (TPP) [class: Quaternary Ammonium Compounds (QACs)], Benzylkonium Chloride (BAC) [class: Quaternary Ammonium Compounds (QACs)], Ethidium Bromide [class: Phenanthridine], Sodium Dodecyl Sulfate (SDS) [class: Organo-sulfate] |
| 79 | BAC0384 | *tehA* | *Escherichia coli* (strain K12) | Chromosome | Tellurium (Te), Proflavin [class: Acridine], Tetraphenylphosphonium (TPP) [class: Quaternary Ammonium Compounds (QACs)], Ethidium Bromide [class: Phenanthridine], Crystal Violet [class: Triarylmethane], Dequalinium [class: Quaternary Ammonium Compounds (QACs)], Methyl Viologen [class: Paraquat] |
| 80 | BAC0385 | *tehB* | *Escherichia coli* (strain K12) | Chromosome | Tellurium (Te), Proflavin [class: Acridine] |
| 81 | BAC0393 | *tolC* | *Escherichia coli* (strain K12) | Chromosome | Phenol [class: Phenolic compounds], Triclosan [class: Phenolic compounds], Sodium Deoxycholate (SDC) [class: Acid], Sodium Cholate [class: Acid], Sodium Taurodeoxycholate [class: Acid], Sodium Dodecyl Sulfate (SDS) [class: Organo-sulfate], Proflavin [class: Acridine], Acriflavine [class: Acridine], Ethidium Bromide [class: Phenanthridine], Tetraphenylphosphonium (TPP) [class: Quaternary Ammonium Compounds (QACs)], Rhodamine 6G [class: Xanthene], Tetraphenylarsonium (TPA) [class: Quaternary Ammonium Compounds (QACs)], Cetrimide (CTM) [class: Quaternary Ammonium Compounds (QACs)], Dequalinium [class: Quaternary Ammonium Compounds (QACs)], Benzylkonium Chloride (BAC) [class: Quaternary Ammonium Compounds (QACs)], Plumbagin [class: Naphthoquinone] |
| 82 | BAC0434 | *ychH* | *Escherichia coli* (strain K12) | Chromosome | Cadmium (Cd), Hydrogen Peroxide (H2O2) [class: Peroxides] |
| 83 | BAC0436 | *ydeI* | *Escherichia coli* (strain K12) | Chromosome | Hydrogen Peroxide (H2O2) [class: Peroxides] |
| 84 | BAC0437 | *ydeO* | *Escherichia coli* (strain K12) | Chromosome | Hydrochloric acid (HCl) [class: Acid], Rhodamine 6G [class: Xanthene], Sodium Dodecyl Sulfate (SDS) [class: Organo-sulfate] |
| 85 | BAC0438 | *ydeP* | *Escherichia coli* (strain K12) | Chromosome | Hydrochloric acid (HCl) [class: Acid] |
| 86 | BAC0445 | *ygiW* | *Escherichia coli* | Plasmid pMR-1 (Shewanella oneidensis (strain MR-1)) | Cadmium (Cd), Tributyltin (TBT) [class: Organo-tin], Hydrogen Peroxide (H2O2) [class: Peroxides] |
| 87 | BAC0447 | *yjaA* | *Escherichia coli* (strain K12) | Chromosome | Cadmium (Cd), Hydrogen Peroxide (H2O2) [class: Peroxides], Hydrochloric acid (HCl) [class: Acid] |
| 88 | BAC0450 | *ymgB/ariR* | *Escherichia coli* (strain K12) | Plasmid pPag3 (Pantoea vagans (strain C9-1) (Pantoea agglomerans (strain C9-1))) | Hydrochloric Acid (HCl) [class: Acid], Hydrogen Peroxide (H2O2) [class: Peroxides] |
| 89 | BAC0451 | *yodD* | *Escherichia coli* (strain K12) | Chromosome | Cadmium (Cd), Hydrogen Peroxide (H2O2) [class: Peroxides], Hydrochloric acid (HCl) [class: Acid] |
| 90 | BAC0452 | *yqjH* | *Escherichia coli* (strain K12) | Chromosome | Iron (Fe), Nickel (Ni) |
| 91 | BAC0457 | *zinT/yodA* | *Escherichia coli* (strain K12) | Chromosome | Cadmium (Cd), Zinc (Zn) |
| 92 | BAC0459 | *zitB/ybgR* | *Escherichia coli* (strain K12) | Chromosome | Zinc (Zn) |
| 93 | BAC0461 | *zntA/yhhO* | *Escherichia coli* (strain K12) | Chromosome | Lead (Pb), Cadmium (Cd), Zinc (Zn) |
| 94 | BAC0462 | *zntR/yhdM* | *Escherichia coli* (strain K12) | Chromosome | Zinc (Zn) |
| 95 | BAC0463 | *znuA/yebL* | *Escherichia coli* (strain K12) | Chromosome | Zinc (Zn) |
| 96 | BAC0464 | *znuB/yebI* | *Escherichia coli* (strain K12) | Chromosome | Zinc (Zn) |
| 97 | BAC0465 | *znuC/yebM* | *Escherichia coli* (strain K12) | Chromosome | Zinc (Zn) |
| 98 | BAC0467 | *zraR/hydH* | *Escherichia coli* (strain K12) | Chromosome | Zinc (Zn) |
| 99 | BAC0468 | *zraS/hydG* | *Escherichia coli* (strain K12) | Chromosome | Zinc (Zn), Lead (Pb) |
| 100 | BAC0469 | *zupT/ygiE* | *Escherichia coli* (strain K12) | Chromosome | Zinc (Zn), Iron (Fe), Cobalt (Co), Nickel (Ni), Copper (Cu), Cadmium (Cd) |
| 101 | BAC0470 | *zur/yjbK* | *Escherichia coli* (strain K12) | Chromosome | Zinc (Zn) |
| 102 | BAC0530 | *phoB* | *Klebsiella pneumoniae*  NTUH-K2044 | Chromosome | Benzylkonium Chloride (BAC) [class: Quaternary Ammonium Compounds (QACs)], Chlorhexidine [class: Biguanides] |
| 103 | BAC0532 | *cpxA* | *Klebsiella pneumoniae* *subsp. pneumoniae* NTUH-K2044 | Chromosome | Hydrogen Peroxide (H2O2) [class: Peroxides], Benzylkonium Chloride (BAC) [class: Quaternary Ammonium Compounds (QACs)], Chlorhexidine [class: Biguanides] |
| 104 | BAC0533 | *cpxR* | *Klebsiella pneumoniae subsp. pneumoniae* NTUH-K2044 | Chromosome | Hydrogen Peroxide (H2O2) [class: Peroxides], Benzylkonium Chloride (BAC) [class: Quaternary Ammonium Compounds (QACs)], Chlorhexidine [class: Biguanides] |
| 105 | BAC0536 | *oxyRkp* | Klebsiella pneumoniae  NTUH-K2044 | Chromosome | Hydrogen Peroxide (H2O2) [class: Peroxides], Sodium Deoxycholate (SDC) [class: Acid], Acriflavine [class: Acridine], Rhodamine 6G [class: Xanthene], Ethidium Bromide [class: Phenanthridine], Sodium Dodecyl Sulfate (SDS) [class: Organo-sulfate] |
| 106 | BAC0540 | *nfsA* | *Escherichia coli* K12 | Chromosome | Chromium (Cr) |
| 107 | BAC0541 | *yieF* | *Escherichia coli* K12 | Chromosome | Chromium (Cr), Vanadium (V), Molybdenum (Mo), Methylene Blue [class: Thiazinium] |
| 108 | BAC0559 | *emrR* | *Escherichia coli* (strain K12) | Chromosome | Carbonyl cyanide 3-chlorophenylhydrazone (CCCP) [class: Hydrazone] |
| 109 | BAC0560 | *marA* | *Escherichia coli* (strain K12) | Chromosome | Cyclohexane [class: Cycloalkane], Diphenyl Ether [class: Phenyl], n-hexane [class: Alkane] |
| 110 | BAC0561 | *marR* | *Escherichia coli* (strain K12) | Chromosome | Cyclohexane [class: Cycloalkane], Diphenyl Ether [class: Phenyl], n-hexane [class: Alkane] |
| 111 | BAC0563 | *acrD* | *Salmonella enterica serovar Typhimurium* | Chromosome | Copper (Cu), Zinc (Zn) |
| 112 | BAC0564 | *actP/yjcG* | *Escherichia coli* (strain K12) | Chromosome | Sodium Glycocholate [class: Acid], Sodium acetate [class: Acetate] |
| 113 | BAC0576 | *arsB* | *Escherichia coli* | Plasmid IncN R46 | Arsenic (As), Antimony (Sb) |
| 114 | BAC0578 | *arsB* | *Acidiphilium multivorum*  (strain DSM 11245 / JCM 8867 / AIU301) | Plasmid pKW301 | Arsenic (As), Antimony (Sb) |
| 115 | BAC0582 | *arsC* | *Escherichia coli* | Plasmid R773 | Arsenic (As), Antimony (Sb) |
| 116 | BAC0583 | *arsC* | *Escherichia coli* | Plasmid IncN R46 | Arsenic (As), Antimony (Sb) |
| 117 | BAC0584 | *arsC* | *Acidiphilium multivorum* (strain DSM 11245 / JCM 8867 / AIU301) | Plasmid pKW301 | Arsenic (As), Antimony (Sb) |
| 118 | BAC0594 | *arsR* | *Escherichia coli* (strain K12) | Chromosome | Arsenic (As) |
| 119 | BAC0596 | *baeR* | *Salmonella Typhimurium* | Chromosome | Copper (Cu), Zinc (Zn), Tungsten (W), Sodium Deoxycholate (SDC) [class: Acid] |
| 120 | BAC0608 | *modE* | *Escherichia coli* (strain K12) | Chromosome | Tungsten (W), Molybdenum (Mo) |
| 121 | BAC0609 | *modA* | *Escherichia coli* (strain K12) | Chromosome | Tungsten (W), Molybdenum (Mo) |
| 122 | BAC0610 | *modB* | *Escherichia coli* (strain K12) | Chromosome | Tungsten (W), Molybdenum (Mo) |
| 123 | BAC0611 | *modC* | *Escherichia coli* (strain K12) | Chromosome | Tungsten (W), Molybdenum (Mo) |
| 124 | BAC0641 | *corA* | *Salmonella typhimurium* (strain LT2 / SGSC1412 / ATCC 700720) | Chromosome | Magnesium (Mg), Cobalt (Co), Nickel (Ni), Manganese (Mn) |
| 125 | BAC0643 | *corB* | *Salmonella typhimurium* (strain LT2 / SGSC1412 / ATCC 700720) | Chromosome | Cobalt (Co), Magnesium (Mg) |
| 126 | BAC0644 | *corD* | *Salmonella typhimurium* (strain LT2 / SGSC1412 / ATCC 700720) | Chromosome | Cobalt (Co), Magnesium (Mg) |
| 127 | BAC0646 | *mdtB* | *Salmonella enterica serovar Typhimurium* | Chromosome | Zinc (Zn) |
| 128 | BAC0647 | *mdtC* | *Salmonella enterica serovar Typhimurium* | Chromosome | Zinc (Zn) |
| 129 | BAC0707 | *chtR* | *Enterococcus faecium* | Chromosome | Chlorhexidine [class: Biguanides] |
| 130 | BAC0707 | *sodB* | *Escherichia coli* K12 | Chromosome | Selenium (Se), Hydrogen Peroxide (H2O2) [class: Peroxides] |
| 131 | BAC0725 | *copA* | *Escherichia coli* K-12 | Chromosome | Copper (Cu) |
